# Single‐cell RNA sequencing reveals reduced intercellular adhesion molecule crosstalk between activated hepatic stellate cells and neutrophils alleviating liver fibrosis in hepatitis B virus transgenic mice post menstrual blood‐derived mesenchymal stem cell transplantation

**DOI:** 10.1002/mco2.654

**Published:** 2024-07-22

**Authors:** Lijun Chen, Yuqi Huang, Ning Zhang, Jingjing Qu, Yangxin Fang, Jiamin Fu, Yin Yuan, Qi Zhang, Hang Li, Zuoshi Wen, Li Yuan, Lu Chen, Zhenyu Xu, Yifei Li, Huadong Yan, Hiromi Izawa, Lanjuan Li, Charlie Xiang

**Affiliations:** ^1^ State Key Laboratory for Diagnosis and Treatment of Infectious Diseases National Clinical Research Center for Infectious Diseases National Medical Center for Infectious Diseases Collaborative Innovation Center for Diagnosis and Treatment of Infectious Diseases The First Affiliated Hospital Zhejiang University School of Medicine Hangzhou China; ^2^ Research Units of Infectious Disease and Microecology Chinese Academy of Medical Sciences Beijing China; ^3^ Department of Respiratory Disease Thoracic Disease Centre The First Affiliated Hospital Zhejiang University School of Medicine Hangzhou China; ^4^ Innovative Precision Medicine (IPM) Group Hangzhou China; ^5^ Department of Cardiology The First Affiliated Hospital Zhejiang University School of Medicine Hangzhou China; ^6^ Infectious Disease Department Shulan (Hangzhou) Hospital Affiliated to Zhejiang Shuren University Shulan International Medical College Hangzhou China; ^7^ Jingu‐Gaien Woman Life Clinic Tokyo Japan; ^8^ Jinan Microecological Biomedicine Shandong Laboratory Jinan China

**Keywords:** hepatic stellate cell (HSC), HSC and neutrophil crosstalk, liver fibrosis in HBV‐Tg mouse, menstrual blood‐derived MSC, single‐cell RNA sequencing

## Abstract

Liver fibrosis can cause hepatitis B virus (HBV)‐associated hepatocellular carcinoma. Menstrual blood‐derived mesenchymal stem cells (MenSCs) can ameliorate liver fibrosis through paracrine. Single‐cell RNA sequencing (scRNA‐seq) may be used to explore the roadmap of activated hepatic stellate cell (aHSC) inactivation to target liver fibrosis. This study established HBV transgenic (HBV‐Tg) mouse model of carbon tetrachloride (CCl_4_)‐induced liver fibrosis and demonstrated that MenSCs migrated to the injured liver to improve serological indices and reduce fibrotic accumulation. RNA‐bulk analysis revealed that MenSCs mediated extracellular matrix accumulation and cell adhesion. Liver parenchymal cells and nonparenchymal cells were identified by scRNA‐seq in the control, CCl_4_, and MenSC groups, revealing the heterogeneity of fibroblasts/HSCs. A CellChat analysis revealed that diminished intercellular adhesion molecule (ICAM) signaling is vital for MenSC therapy. Specifically, *Icam1* in aHSCs acted on *Itgal*/*Itgb2* and *Itgam*/*Itgb2* in neutrophils, causing decreased adhesion. The expression of *Itgal*, *Itgam*, and *Itgb2* was higher in CCl_4_ group than in the control group and decreased after MenSC therapy in neutrophil clusters. The *Lcn2*, *Pglyrp1*, *Wfdc21*, and *Mmp8* had high expression and may be potential targets in neutrophils. This study highlights interacting cells, corresponding molecules, and underlying targets for MenSCs in treating HBV‐associated liver fibrosis.

## Graphical abstract

MenSCs improved serological indices in HBV‐Tg mouse model of CCl_4_‐induced liver fibrosis. The scRNA‐seq identified 12 main cell populations in control, CCl_4_, and MenSC groups in HBV‐Tg mice livers. Reducing ICAM crosstalk between aHSCs and neutrophils is one of the underlying mechanisms. This study highlights interacting target cells and corresponding molecules for MenSCs in treating HBV‐associated liver fibrosis.

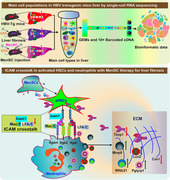

## 1 INTRODUCTION

Liver diseases, including liver cirrhosis, viral hepatitis, and hepatocellular carcinoma, account for over 2 million deaths annually, and there are over 90 million hepatitis B virus (HBV) carriers in China.^1^, ^2^ Currently, there are no better treatments for middle‐ and end‐stage chronic liver diseases (CLDs) other than orthotopic liver transplantation.^3^ Liver fibrosis is an inevitable course of various CLDs and involves highly dynamic and complex processes.^4^, ^5^ Accompanied by continuous liver damage, the excessive accumulation of the extracellular matrix (ECM) causes liver fibrosis and physiological and pathological changes,^6^, ^7^ including the recruitment of various inflammatory and immune cells, barrier damage to liver sinusoidal endothelial cells (LSECs), activation of hepatic stellate cells (HSCs), and necrosis of hepatocytes.^8^, ^9^ Hepatic myofibroblasts are the main cells producing the ECM and may originate from HSCs, portal fibroblasts (PFs), or mesothelial cells.^10^, ^11^ Cell fate mapping and deep phenotyping analyses have indicated that activated hepatic stellate cells (aHSCs) and activated portal fibroblasts (aPFs) account for more than 90% of collagen‐producing cells in experimental animal models of liver fibrosis.^12^ Generally, PFs play an important role in bile duct ligation‐induced liver fibrosis, and HSCs play a vital role in carbon tetrachloride (CCl_4_)‐induced liver fibrosis.^13^ HSC activation is a key event in the progression of liver fibrosis.^14^, ^15^ In advanced hepatic fibrosis, interleukin (IL)‐17^+^ neutrophils mediate the activation of HSCs by regulating the ratio of T helper (Th)‐17 and T regulatory cells, releasing transforming growth factor β1 (TGF‐β1), which causes the deposition of type I collagen (Col‐I), triggering the immune response disorder.^16^ Understanding how neutrophils interact with HSCs may reveal the molecular mechanisms underlying neutrophil and ECM regulation during liver fibrosis. Neutrophils are an important component of leukocytes as the first line of defense against invading pathogens.^17^, ^18^ Moreover, neutrophils are vital participants in the processes that affect liver injury and increase the risk of infection and susceptibility to scathing organs in acute‐on‐chronic liver failure^19^ and liver cirrhosis.^20^

Single‐cell RNA sequencing (scRNA‐seq) is changing our understanding of the cellular and molecular mechanisms underlying liver diseases.^21^, ^22^ scRNA‐seq has enabled the study of various cell subpopulations, molecular processes, and interactions among different cell types in liver parenchymal cells, such as hepatocytes, cholangiocytes, and liver nonparenchymal cells (NPCs), including LSECs, HSCs, Kupffer cells, myofibroblasts/fibroblasts, and other immune cells.^23^, ^24^, ^25^, ^26^ Advances in single‐cell phenotyping have allowed the cellular classification of the origins of myofibroblasts during fibrosis under different disease conditions and the corresponding functional in‐depth dissections.^27^, ^28^ Activation from quiescent HSCs (qHSCs) to aHSCs has been extensively studied in liver fibrosis models using scRNA‐seq.^25^, ^29^, ^30^, ^31^ However, the role of HSC inactivation, from aHSCs to inactivated HSCs (iHSCs), has not been explored to the same extent at the scRNA‐seq level, with a major challenge being the evaluation of HSC inactivation status.^32^ Rosenthal et al.^33^ indicated that HSCs, including qHSCs, aHSCs, and iHSCs, are heterogeneous based on their scRNA‐seq expression profiles in the livers of nonalcoholic steatohepatitis (NASH) *foz/foz* mice. Liu et al.^34^ found that GATA binding factor 6 and peroxisome proliferator‐activated receptor‐γ (PPARγ) are pivotal transcription factors, using the small RNA knockdown method to inactivate HSCs in liver fibrosis in humans and mice. The signaling pathways and corresponding molecular mechanisms involved in the inactivation of aHSCs into iHSCs should be explored at the single‐cell level.

Neutrophils and HSCs interact through the production of granulocyte‐macrophage colony‐stimulating factor and IL‐15 in aHSCs to prolong neutrophil survival rates, which in turn promote a positive cycle of fibrosis in experimental steatohepatitis.^35^ Intercellular adhesion molecules (ICAMs/cluster of differentiation (CD)‐54) are important components of cell surface glycoproteins involved in the adhesion, immune response, and intracellular signaling. ICAM1 is the most extensively studied ligand and is mainly expressed in leukocytes, endothelial cells, fibroblasts, and epithelial cells.^36^ ICAM1 binds to the heterodimer of leukocyte function associated antigen‐1 (LFA‐1) to form a complex of integrin αL/β2 subunits (*Itgal*/*Itgb2*, known as CD11a/CD18) and the heterodimer of macrophage‐1 antigen (MAC‐1) to form a complex of integrin αM/β2 subunits (*Itgam*/*Itgb2*, known as CD11b/CD18).^37^ Integrins can work synergistically with the actin cytoskeleton to facilitate cell adhesion, migration, or invasion to maintain the integrity of epithelial tissues.^38^ ICAM1 further affects the induction of patient‐derived endothelial cells, and *Itgb2* increases neutrophil adhesion and migration.^39^

Mesenchymal stem cells (MSCs), a major type of adult stem cells, are derived from the tissue mesoderm.^40^, ^41^ MSCs have immunomodulatory properties for tissue repair, making them therapeutic agents with great potential for the treatment of liver fibrosis and cirrhosis.^42^, ^43^, ^44^ Menstrual blood‐derived mesenchymal stem cells (MenSCs) are tissue‐specific MSCs exfoliated from the endometrium during the menstrual cycle in women.^45^, ^46^ Like other MSCs, MenSCs improve liver fibrosis through paracrine effects by inhibiting HSC proliferation.^47^ Owing to the lack of ideal HBV mouse models, the effects of MenSCs on HBV liver fibrosis and their molecular mechanisms have been unclear. Recently, Tang et al.^48^ used HBV transgenic (HBV‐Tg) mice to confirm that the signal transducer and activator of transcription 3 (STAT3) is a key factor in promoting the activation of liver inflammation induced by HBV. In addition, by comparing HBV‐Tg mice of different ages with liver samples from patients with chronic hepatitis B (CHB), the study demonstrated similarities in the immunological/inflammatory pathways between the HBV‐Tg mice and CHB patients. Thus, HBV‐Tg mice may be suitable for mimicking HBV infection.

**FIGURE 1:**
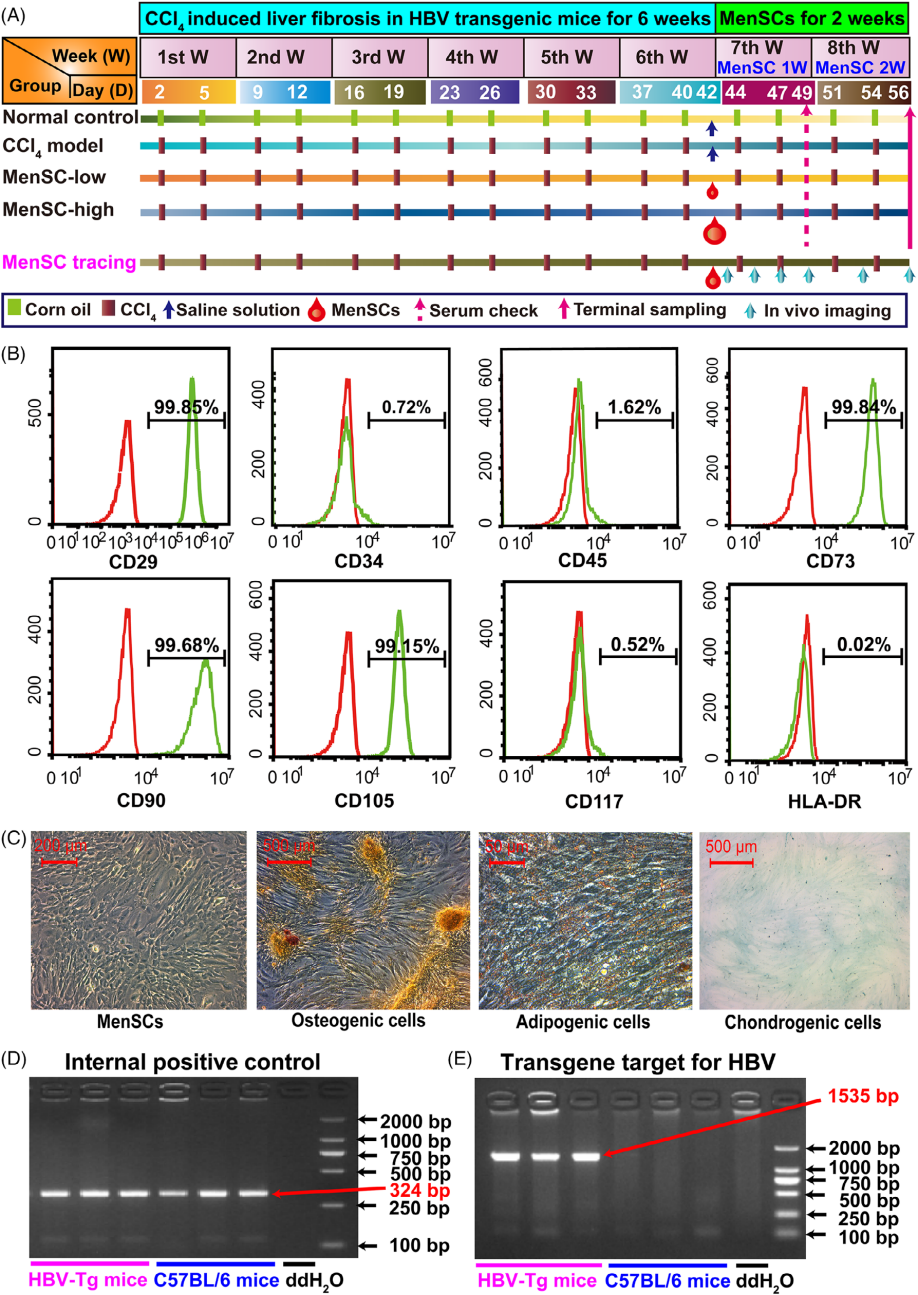
Schematic representation of the experimental process in the different studied groups; identification of menstrual blood‐derived MSCs (MenSCs) and hepatitis B virus transgenic (HBV‐Tg) mice. (A) Schematic diagram for CCl_4_‐induced liver fibrosis and MenSC transplantation within 2 weeks in HBV‐Tg mice. (B) Flow cytometry was performed to identify the immunophenotyping of MenSCs by the expression of CD29, CD34, CD45, CD73, CD90, CD105, CD117, and HLA‐DR. (C) MenSCs differentiated into osteogenic, adipogenic, and chondrogenic cells. The expression of internal positive control (D) and HBV transgene targets (E) were checked in HBV‐Tg mice and C57BL/6 mice using RT‐PCR.

In this study, the integrated therapeutic effects of MenSC transplantation were verified in HBV‐Tg mouse liver fibrosis composite models (Figure 1A). The scRNA‐seq analyses revealed 12 major cellular clusters, including LSECs, fibroblasts/HSCs, cholangiocytes, hepatocytes, plasma cells, B cells, dendritic cells (DCs), Kupffer cells, T cells, natural killer cells (NKs), neutrophils, and mast cells in mouse liver in the control, CCl_4_, and MenSC groups. The fibroblasts/HSCs populations were reclustered and analyzed to uncover the unique characteristics of aHSCs and iHSCs. The ICAM pathway was identified as a vital factor in the interactions between aHSCs and neutrophils with CellChat analyses. We found that menstrual blood‐derived MSCs significantly relieve liver fibrosis in HBV‐Tg mice, and provide, for the first time, primal data on the use of MenSCs to treat HBV‐associated liver fibrosis in HBV‐Tg mice. We also uncovered the cell–cell adhesion between aHSCs and neutrophils and highlighted the underlying molecular mechanisms via ICAM crosstalk.

## 2 RESULTS

### 2.1 Identification of MenSCs and HBV transgenic mice

The expression of CD29, CD73, CD90, and CD105 was positive, whereas that of CD34, CD45, CD117, and human leukocyte antigen‐DR (HLA‐DR) was negative (Figure 1B). MenSCs displayed a spindle‐ and fibroblast‐like shape and successfully differentiated into osteogenic, adipogenic, and chondrogenic cells under the appropriate conditions (Figure 1C).

The average of hepatitis B surface antigen (HBsAg) remained almost the same in each group of enrolled mice on day 1 (Figure S1A). C57BL/6 mice and HBV‐Tg mice have no phenotypic variations (Figure S1B). According to the polymerase chain reaction (PCR) result, both the HBV‐Tg mice and C57BL/6 mice were positive for the expression of the internal positive control (Figure 1D). The HBV‐Tg mice expressed the HBV transgene target (size 1535 bp); however, the C57BL/6 mice did not express HBV DNA (Figure 1E), which further confirmed the establishment of HBV‐Tg mice.

### 2.2 MenSCs migrated into injured livers and improved serological indicators in HBV‐Tg mouse liver fibrosis

**FIGURE 2:**
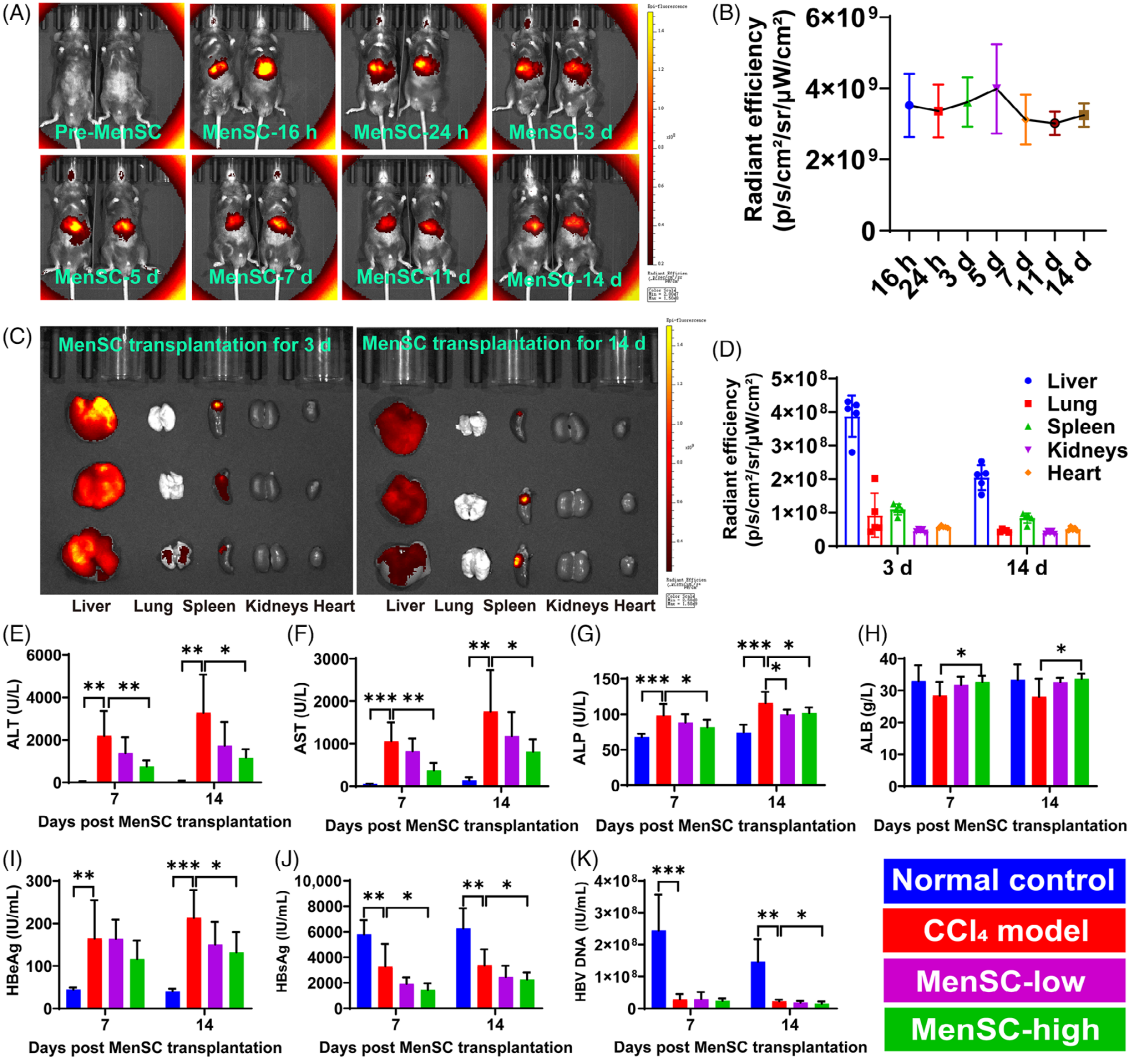
MenSCs migrated into injured livers and improved serological indicators in HBV‐Tg liver fibrosis composite model mice. The fluorescence existed at high intensity (A) within 14 days (before the MenSC injections and 16 h, 24 h, 3 days, 5 days, 7 days, 11 days, and 14 days) of MenSC transplantation by in vivo imaging, and its relative fluorescence intensity (B). Dissected liver, lung, spleen, kidneys, and heart tissues were detected by the fluorescence intensity at 3 and 14 days after MenSC transplantation (C), and the average radiation efficiency of imaging value was presented (D). The liver functions of ALT (E), AST (F), ALP (G), and ALB (H) were detected at 7 and 14 days post MenSC transplantation in the normal control group, CCl_4_ model group, MenSC‐low group, and MenSC‐high group. Similarly, hepatitis B‐related indicators in the serum HBeAg (I), HBsAg (J), and HBV DNA (K) were checked in HBV‐Tg mouse livers. Asterisks (*) marked the significant differences (*p < 0.05, **p < 0.01, ***p < 0.001). Error bars indicated by standard deviation (SD).

In vivo imaging results showed that within 14 days of MenSC transplantation, fluorescence existed at a high intensity (Figure 2A), and on day 5 post MenSC transplantation, the relative fluorescence intensity (*n* = 5) showed the strongest value (Figure 2B). By detecting the fluorescence intensity of the dissected liver, lung, spleen, kidneys, and heart, we found that MenSCs were mainly concentrated in the liver (Figure 2C), and the average radiation efficiency of the mouse liver imaging on 3 days after transplantation was higher than that 14 days (Figure 2D), which was consistent with the in vivo imaging data (Figure 2B). Thus, MenSCs mainly migrated into the injured liver and lasted for at least 2 weeks.

Spleen injections (Figure S1C) and surgical sutures (Figure S1D) for MenSC transplantation were administered. The HBV‐Tg mouse livers in the control group were glossy, but those in the CCl_4_ group sustained injury and produced granular fibrosis (Figure S1E–H). An analysis of body weight (Figure S1I) and normalized body weight (Figure S1J) revealed no significant differences among the CCl_4_, MenSC‐low, and MenSC‐high groups. Most serological indicators in the MenSC‐low group showed remission trends compared with in the CCl_4_ model group. High doses of MenSCs significantly improved alanine aminotransferase (ALT) (Figure 2E), aspartate aminotransferase (AST) (Figure 2F), alkaline phosphatase (ALP) (Figure 2G), and albumin (ALB) (Figure 2H) levels on days 7 and 14 post MenSC transplantation. We studied hepatitis B‐related indicators in the sera of HBV‐Tg mice, including hepatitis B e antigen (HBeAg) (Figure 2I), HBsAg (Figure 2J), and HBV DNA (Figure 2K). The serum HBeAg level in the MenSC‐high group was significantly lower than that in the CCl_4_ model group in MenSC 2 W (Figure 2I). Compared with those in the control group, HBsAg levels in the CCl_4_, MenSC‐low, and MenSC‐high groups were reduced (Figure 2J). This result indicated that mouse hepatocytes were damaged, causing a decrease in the expression of HBsAg. The damaged mouse hepatocytes were unable to express HBsAg, and the ability to express HBV serological indexes was reduced. This indirectly proves that the CCl_4_‐induced liver fibrosis models indeed affected the function of hepatocytes. After MenSC transplantation, HBsAg levels in low‐dose and CCl_4_ model groups for both MenSC 1 W and MenSC 2 W did not differ significantly. The HBsAg levels in the high‐dose group were significantly lower than those in the CCl_4_ model group for both MenSC 1 W and MenSC 2 W (Figure 2J). The HBV DNA level in the CCl_4_ group was significantly lower than that in the control group (Figure 2K), indicating that damaged hepatocytes reduced the capacity for expressing HBV DNA after CCl_4_ sustained injury. Compared with that in the CCl_4_ group, the expression of HBV DNA in the MenSC‐high group was significantly reduced in 2 weeks after MenSC transplantation (Figure 2K).

### 2.3 MenSCs reduced pathological indicators by reducing collagen fiber in HBV‐Tg mice with liver fibrosis

**FIGURE 3:**
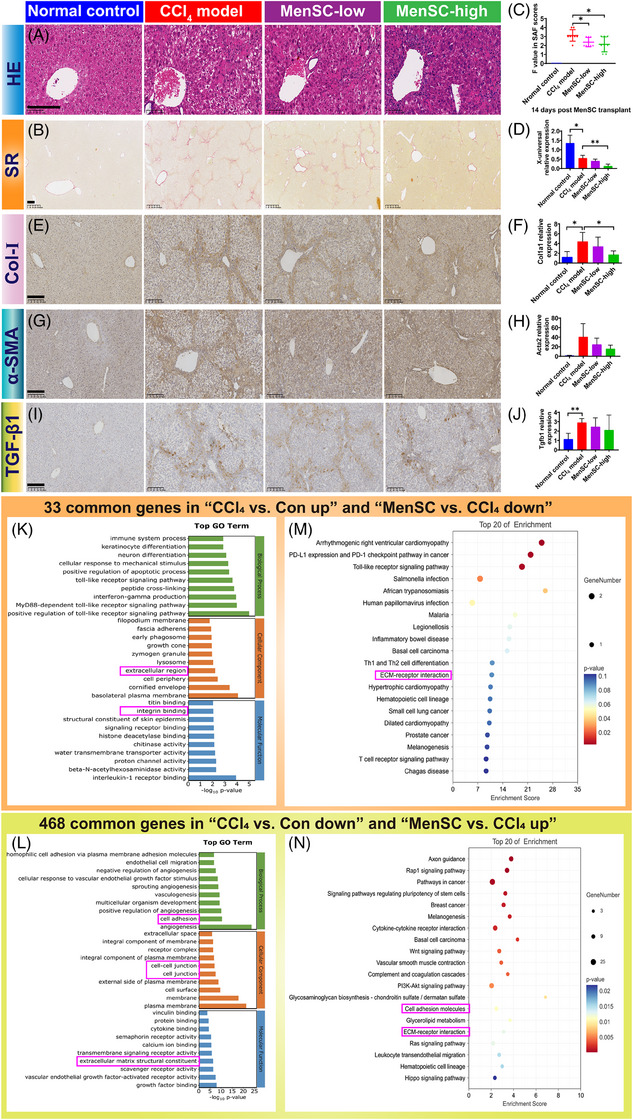
MenSCs reduce collagen fiber and mediate ECM accumulation and cell adhesion to ameliorate HBV‐related liver fibrosis. Representative photomicrographs of liver sections stained with HE staining (A) and SR staining (B) at 14 days post MenSC transplantation in the normal control group, CCl_4_ model group, MenSC‐low group, and MenSC‐high group; scale bar = 200 µm. (C) The liver fibrosis score (SAF) of experimental animals was divided into stages of fibrosis 0−4 (F0−4), and these data were statistically analyzed. (D) HBV‐DNA expression in liver tissue by qRT‐PCR. The expression of Col‐I (E) by immunohistochemistry (IHC) and *Col1a1* (F) by qRT‐PCR was examined. The expression of α‐SMA (G) by IHC and *Acta2* (H) by qRT‐PCR was examined. The expression of TGF‐β1 showed in IHC (I) and *Tgfb1* (J) by qRT‐PCR. According to the RNA‐bulk analysis, the top‐30 GO terms based on the 33 common genes (K) and 468 common genes (L). The top‐20 KEGG results based on the 33 common genes (M) and 468 common genes (N). Asterisks (*) marked the significant differences (*p < 0.05, **p < 0.01). Error bars indicated by SD.

Referring to the steatosis activity fibrosis (SAF) scoring system,^49^ the pathological scores of the livers were evaluated based on the hematoxylin and eosin (HE) and Sirius red (SR) staining (Table S1). The CCl_4_, MenSC‐low, and MenSC‐high groups exhibited hepatic lobular inflammation, hepatocyte hyaline degeneration, ballooning degeneration, bile duct proliferation, and necrosis (Table S1 and Figure 3A,B). Compared with the CCl_4_ group, there was a reduction in hepatic lobular inflammation and hyalinization in the high‐dose group (Figure 3A). After transplanting MenSCs into fibrotic mice for 2 weeks, the collagen fiber area was reduced (Figure 3B). Compared with the fibrosis scores of the CCl_4_ model group (3.1 ± 0.6), those of the MenSC‐low (2.4 ± 0.5) and MenSC‐high (2.1 ± 0.8) groups were significantly reduced (Figure 3C). A quantitative real‐time PCR (qRT‐PCR) analysis further revealed that HBV DNA (named X‐universal) expression in the liver tissue showed a significant decreasing trend in MenSC‐high group compared with CCl_4_ model group (Figure 3D). The expression of Col‐I (Figure 3E), as shown by immunohistochemistry (IHC), and *Col1a1* (Figure 3F), as shown by qRT‐PCR, was significantly higher in the CCl_4_ model group than in the control group. After high‐dose MenSC transplantation, the expression of *Col1a1* (Figure 3F) significantly decreased in MenSC 2 W. The expression of α‐smooth muscle actin (α‐SMA; *Acta2*) (Figure 3G), as shown by IHC, and *Acta2* (Figure 3H), as shown by qRT‐PCR, was increased in CCl_4_ model group compared with that control group. The difference in *Acta2* expression among the four groups was not significant (Figure 3H); however, the observed trend was similar to that of *Col1a1*. The expression of TGF‐β1 showed no significant difference in the IHC (Figure 3I) and qRT‐PCR (Figure 3J). Thus, MenSCs reduced fibrotic accumulation independent of TGF‐β1 signal, as shown by qRT‐PCR analysis.

According to the RNA‐bulk analysis, there were 33 common genes in “CCl_4_ versus Con up” and “MenSC versus CCl_4_ down” and 468 common genes in “CCl_4_ versus Con down” and “MenSC versus CCl_4_ up” (Table S2 and Figure S2A,B). Based on the common elements, Kyoto Encyclopedia of Genes and Genomes (KEGG) pathways were classified as infectious and immune‐related diseases (Figure S2C,D). Extracellular region/ECM structural constituents were in the top‐30 Gene Ontology (GO) Terms (Figure 3K,L) and ECM–receptor interaction was in the top‐20 KEGG result (Figure 3M,N). Cell adhesion (integrin binding or cell–cell junction) was a common top‐30 GO Term (Figure 3K,L). Therefore, MenSCs may mediate ECM accumulation and cell adhesion during HBV‐associated liver fibrosis.

### 2.4 scRNA‐seq identified liver multiple cell populations in HBV‐Tg mice with liver fibrosis

**FIGURE 4:**
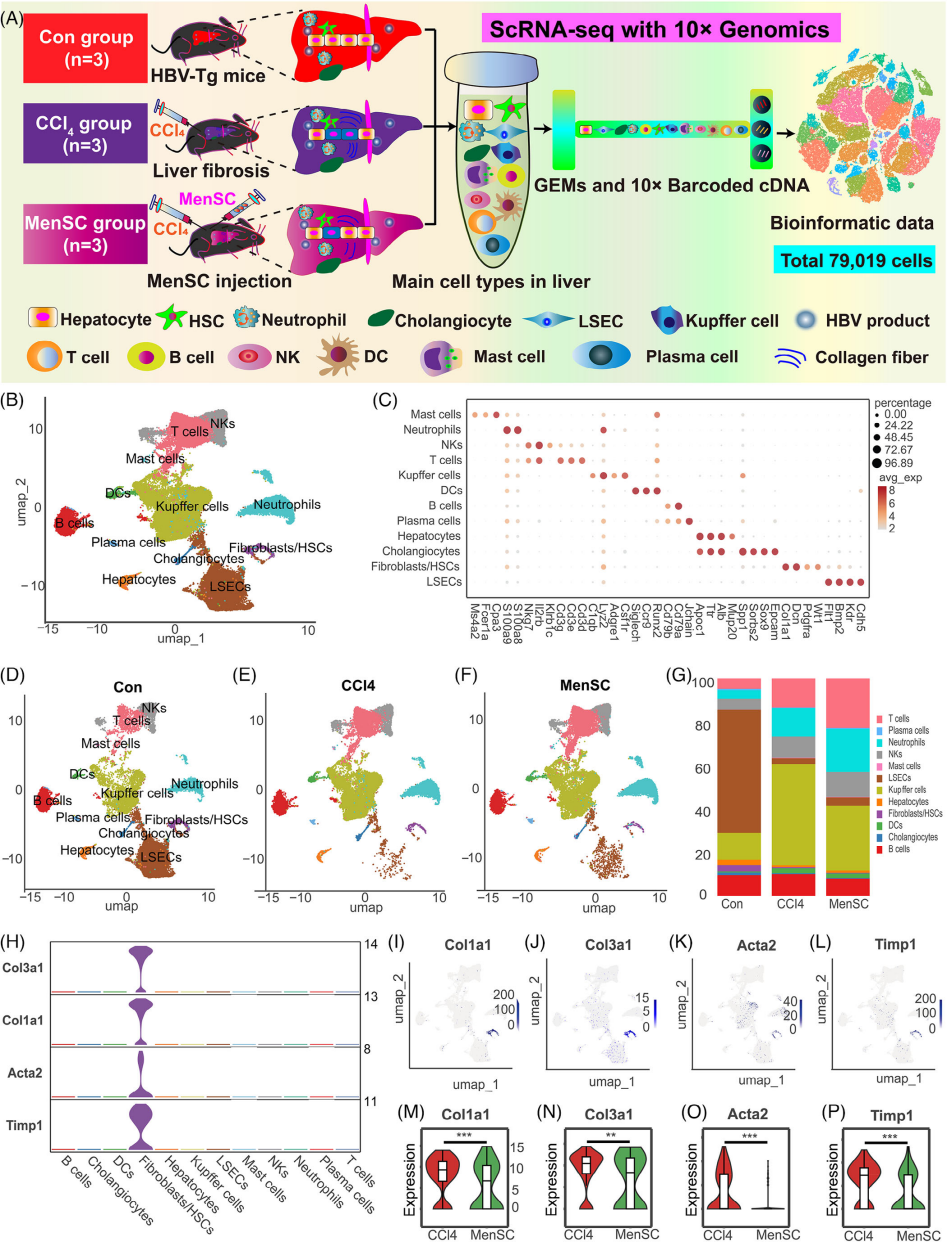
The scRNA‐seq identified 12 main cell populations for liver NPCs and partial liver parenchymal cells in HBV‐Tg mice. (A) Schematic diagram for scRNA‐seq with 10× Genomics. A total of 79,019 cells were included in the analysis, including 25,714 cells in the control group; 25,726 cells in the CCl_4_ group; and 27,579 cells in the MenSC group. (B) Based on the expression of known gene markers, 12 major cell types were grouped by unsupervised clustering analysis of UMAP. (C) A dotplot of 12 major populations along with the corresponding gene markers: LSECs, fibroblasts/HSCs, cholangiocytes, hepatocytes, plasma cells, B cells, DCs, Kupffer cells, T cells, NKs, neutrophils, and mast cells. The main cell populations of UMAP in the Con group (D), CCl_4_ group (E), and MenSC group (F). (G) The cell proportion for 12 major clusters in the Con group, CCl_4_ group, and MenSC group. (H) The expression of well‐known liver fibrosis‐inducing genes (*Col1a1*, *Col3a1*, *Acta*2, and *Timp1*) with cell type‐specific distribution was mainly concentrated in the population of fibroblasts/HSCs. An overview of Feature Plot for *Col1a1* (I), *Col3a1* (J), *Acta2* (K), and *Timp1* (L). The relative expression of *Col1a1* (M), *Col3a1* (N), *Acta2* (O), and *Timp1* (P) were compared between the CCl_4_ group and MenSC group.

A schematic of scRNA‐seq using 10× Genomics is presented in Figure 4A. In total, 81,902 cells were collected. After passing quality control and filtering, 79,019 cells were included in the analysis: 25,714 in the control group, 25,726 in the CCl_4_ group, and 27,579 in the MenSC group. Initial visual diagrams of t‐distributed Stochastic Neighborhood Embedding (t‐SNE) (Figure S3A) and Uniform Mobility Approximation and Projection (UMAP) (Figure S3B) were generated, and 32 cell clusters were identified. The Gene_Counts_RNA profile was presented for 32 cell clusters (Figure S3C). During the analysis of the cell samples, cells that had a mitochondrial gene content exceeding 25% were removed from the dataset (Figure S3D). An clustering analysis of UMAP (Figure 4B) was performed based on the expression of known markers referred to related studies.^29^, ^30^, ^50^, ^51^, ^52^, ^53^, ^54^ Twelve major cell types were grouped along with the corresponding gene markers: LSECs (*Cdh5, Kdr, Bmp2, Flt1*), fibroblasts/HSCs (*Wt1, Pdgfra, Dcn, Col1a1*), cholangiocytes (*Epcam, Sox9, Sorbs2, Spp1*), hepatocytes (*Alb, Ttr, Mup20, Apoc1*), plasma cells (*Jchain*), B cells (*Cd79a, Cd79b*), DCs (*Runx2, Ccr9, Siglech*), Kupffer cells (*Csf1r, Adgre1, Lyz2, C1qb*), T cells (*Cd3d, Cd3e, Cd3g*), NKs (*Il2rb, Nkg7, Klrb1c*), neutrophils (*S100a8, S100a9*), and mast cells (*Cpa3, Fcer1a, Ms4a2*) (Table S3 and Figures S3E and 4C). Feature plots of representative markers for each cluster are presented in Figure S3F–Q. A heatmap with the top‐10 markers of differentially expressed genes (DEGs) in 12 cell types is presented (Figure S4A), which may act as potential markers for future studies. The cluster analysis showed that the main cell populations in the normal control and liver fibrosis groups (CCl_4_ and MenSC groups) were similar, but their proportions differed after CCl_4_‐induced liver fibrosis and following MenSC transplantation (Figures S4B and 4D–G). Compared with those in the control group, there were significant changes in LSECs, Kupffer cells, T cells, and neutrophils in the fibrosis groups. LSECs were drastically reduced in CCl_4_ and MenSC groups compared with control group (Figure 4G). Conversely, the proportions of Kupffer cells, T cells, and neutrophils increased significantly, suggesting a proinflammatory state induced by CCl_4_. The heatmap revealed that well‐known collagen‐producing genes (*Col1a1*, *Col1a2*, and *Col3a1*) were specifically expressed in fibroblasts/HSCs subpopulations (Figure S4A). As shown in Figure 4H, the expression of classic fibrosis‐inducing genes (*Col1a1, Col3a1, Acta2*, and *Timp1*) with a cell type‐specific distribution was mainly concentrated in the population of fibroblasts/HSCs. Feature plots for *Col1a1* (Figure 4I), *Col3a1* (Figure 4J), *Acta2* (Figure 4K), and *Timp1* (Figure 4L) further indicate that collagenous fibers are predominant in the populations of fibroblasts/HSCs. The relative expression of *Col1a1* (Figure 4M), *Col3a1* (Figure 4N), *Acta2* (Figure 4O), and *Timp1* (Figure 4P) was lower in the MenSC group than in the CCl_4_ group, which was anastomotic with the IHC and qRT‐PCR results of Col‐I and α‐SMA in vivo. Therefore, these results further verified the therapeutic effects of MenSCs in reducing liver fibrosis in HBV‐Tg mice at the scRNA‐seq level.

### 2.5 ScRNA‐seq identified the heterogeneity of fibroblasts/HSCs and key molecules, from aHSCs to iHSCs

**FIGURE 5:**
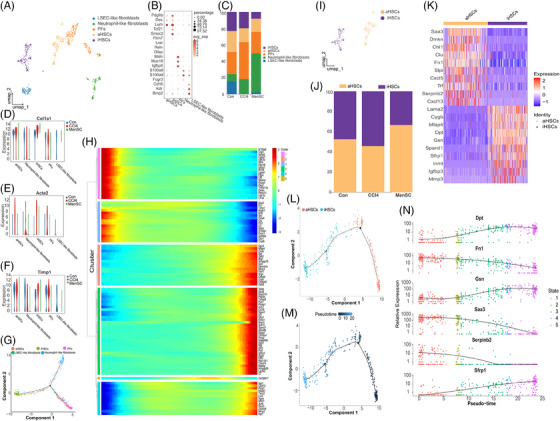
ScRNA‐seq identified the heterogeneity of fibroblasts/HSCs and key molecules from aHSCs into iHSCs in HBV‐Tg mice liver. (A) The fibroblasts/HSCs were identified and integrated for five subpopulations, including aHSCs, iHSCs, PFs, neutrophil‐like fibroblasts, and LSEC‐like fibroblasts by UMAP. (B) A dotplot of five major clusters along with the corresponding gene markers: aHSCs (*Pdgfrb*, *Des*, *Lum*); iHSCs (*Tcf21*, *Smoc2*, *Fbln7*); PFs (*Msln*, *Muc16*, *Igfbp5*); neutrophil‐like fibroblasts (*S100a8*, *S100a9*, *Fcgr3*); and LSEC‐like fibroblasts (*Cdh5*, *Kdr*, *Bmp2*). (C) The relative proportion of each cell type in the Con group, CCl_4_ group, and MenSC group. The relative expression profiles in *Col1a1*, *Acta2*, *Timp1* in the fibroblasts/HSCs population were investigated. The expression of *Col1a1* (D), *Acta2* (E), *Timp1* (F) were significant reduced in MenSC group in contrast to the CCl_4_ model group. (G) Different cell types in the Monocle2. (H) Six sub‐subpopulations and their relative genes with regard to the time process by Monocle2 for clusters. (I) The aHSCs/iHSCs were displayed by UMAP. (J) The relative proportion of aHSCs and iHSCs in the Con group, CCl_4_ group, and MenSC group. (K) The heatmap of top 10 markers of DEGs in aHSCs and iHSCs. (L) aHSCs and iHSCs in the Monocle2. (M) Pseudotime analysis in aHSCs and iHSCs. (N) With the time course, the most upping genes for the aHSCs into iHSCs were *Dpt*, *Gsn*, and *Sfrp1*, and the most downing genes *Fn1*, *Serpinb2*, and *Saa3*.

Unsupervised clustering analyses using UMAP (Figure S5A) identified nine cell populations in fibroblasts/HSCs. It was difficult to define clustered single cells by classic markers including *Col1a1*, *Acta2*, and *Timp1* (Figure S5B) in the scRNA‐seq database. Drawing on other related studies,^13^, ^25^, ^29^, ^33^, ^50^ we identified five subpopulations: aHSCs (*Pdgfrb, Des, Lum*), iHSCs (*Tcf21, Smoc2, Fbln7*), PFs (*Msln, Muc16, Igfbp5*), neutrophil‐like fibroblasts (*S100a8, S100a9, Fcgr3*), and LSEC‐like fibroblasts (*Cdh5, Kdr, Bmp2*) (Table S4 and Figure 5A,B). Representative markers (*Lum, Smoc2, Msln, S100a8, Bmp2*) for each cluster with a Feature Plot are presented in Figure S5C–G. The relative proportions of each cell type in the three groups are shown (Figures 5C and S5H). The heatmap of the top‐10 markers of DEGs in fibroblasts/HSCs subpopulation is shown (Figure S5I). Some classic markers of qHSCs, including lecithin retinol acyltransferase (*Lrat*), reelin (*Reln*), and glial fibrillary acid protein (*Gfap*), were not expressed in the fibroblast/HSC subpopulation (Figure 5B). These results prove that HBV has a huge impact on the heterogeneity of fibroblasts/HSCs. The expression of *Col1a1* (Figure 5D), *Acta2* (Figure 5E), and *Timp1* (Figure 5F) in fibroblasts/HSCs was reduced in the MenSC group compared with that in the CCl_4_ group. Monocle2 pseudotime results showed that aHSCs were predominant in stage 1 and stage 3, iHSCs were predominant in stage 3 (Figures 5G and S5J–L). Among these genes, with the time course, the most upregulated genes in the fibroblast/HSC proportions were *Dpt* and *Gsn*, and the most downregulated genes were *Crip, Dmkn, Igfbp5*, and *Saa3* (Figure S5M). Additionally, relative gene expression of six sub‐subpopulations with respect to time is shown (Figure 5H).

In the following analyses, we characterized the changes in aHSCs and iHSCs proportions and their transcriptional profiles (Figure 5I,J). The heatmap showing the top‐10 markers of DEGs in aHSCs and iHSCs is presented (Figure 5K). Monocle2 results showed that aHSCs can progress towards iHSCs according to the pseudotime (Figure 5L,M and S5N). With the time course, the most upregulated genes for the aHSCs into iHSCs were *Dpt, Gsn*, and *Sfrp1*, and the most downregulated genes were *Fn1*, *Serpinb2*, and *Saa3* (Figure 5N). Additionally, their relative gene expression with respect to time in the clusters is shown in Figure S5O. According to the relative expression trend and top‐10 DEGs of aHSCs/iHSCs (Figure 5K), liver fibrosis may be reduced by increasing the expression of *Dpt* and *Gsn* and reducing the expression of *Saa3*. Therefore, these genes may be potential therapeutic targets.

### 2.6 Reduced ICAM crosstalk of aHSCs and neutrophils was responsible for ameliorating liver fibrosis after MenSC transplant

**FIGURE 6:**
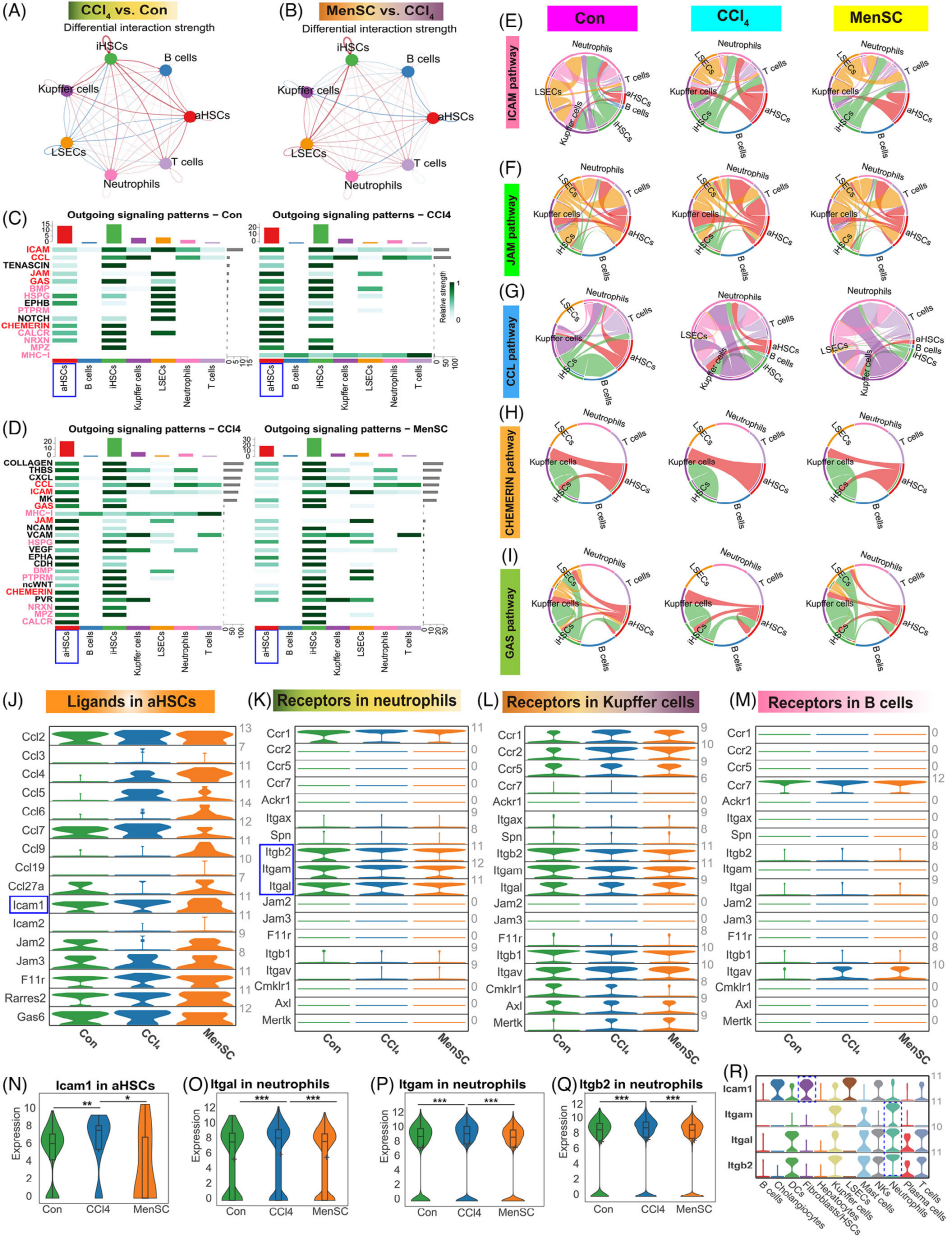
Reduced ICAM crosstalk of aHSCs and neutrophils is responsible for ameliorating liver fibrosis after MenSC transplant by scRNA‐seq analysis. The relationship on aHSCs, iHSCs, T cells, B cells, Kupffer cells, neutrophils, and LSECs in CCl_4_ group versus Con group (A) and MenSC group versus CCl_4_ group (B) by CellChat to expound the aHSCs/iHSCs interaction with other cells. The aHSCs may interact with Kupffer cells, neutrophils and B cells. The output signal of aHSC is higher in the CCl_4_ groups than the Con group and the MenSC group. Among these, 15 signal pathways were stronger than Con group in CCl_4_ group (C), 23 signals were weaker than CCl_4_ group in MenSC group (D). Five common signal channels include ICAM, JAM, CCL, chemerin, and GAS (marked with red font). The chord diagram to further confirm the ICAM pathway (E), JAM pathway (F), CCL pathway (G), CHEMERIN pathway (H), and GAS pathway (I). (J) The aHSCs are output the expression of ligands in five signal pathways in Con, CCl_4_, and MenSC groups. The expression of receptors in five signal pathways in groups of the Con, CCl_4_, and MenSC groups in neutrophils (K), Kupffer cells (L), and B cells (M). ICAM signaling pathway can interact *Icam1* in aHSCs to target the *Itgal*, *Itgam*, and *Itgb2* in neutrophils. (N) The expression of *Icam1* in the aHSCs population was significantly increased in CCl_4_ group in contrast to Con group and MenSC group. Similarly, the expression of the *Itgal* (O), *Itgam* (P), and *Itgb2* (Q) in neutrophils was significantly higher in the CCl_4_ group when compared with the Con group and MenSC group. (R) *Icam1* (blue dotted line) was high expressed in fibroblasts/HSCs, and *Itgal*, *Itgam*, and *Itgb2* (blue dotted line) were high expressed in neutrophils.

The *Icam1* expression for aHSC (LX‐2 cells stimulated with recombinant TGF‐β1) by coculturing with MenSC in vitro (Figure S5P,Q) was assessed. Although the relative expression of *Icam1* was reduced by qRT‐PCR (Figure S5R, 1.02 ± 0.23 in LX‐2 group and 0.78 ± 0.28 in coculture group), however, there are no statistical discrepancy. To further examine the interactions among aHSCs, iHSCs, and other cells, we used CellChat to investigate the relationship between aHSCs, iHSCs, T cells, B cells, Kupffer cells, neutrophils, and LSECs in the CCl_4_ versus control groups (Figure 6A) and MenSC versus CCl_4_ groups (Figure 6B). The strength of the aHSC‐exported signals was significantly increased in neutrophils, Kupffer cells, and B cells in the CCl_4_ group compared with that in the control group (Figure 6A), while the signal strength of the MenSC group significantly decreased after the MenSC therapy (Figure 6B). Further research found that 15 signaling pathways were stronger in the CCl_4_ group than in the control group (Figures S6A and 6C), while 23 signals were weaker in the MenSC group than in the CCl_4_ group (Figures S6B and 6D). Although some common pathways (marked with pink font in Figure 6C,D) were found to be consistent, but there are either no expression or no signals targeting neutrophils/Kupffer cells/B cells. By further contrast, five signal channels were selected (marked with red font in Figure 6C,D), including ICAM, junctional adhesion molecule (JAM), C–C motif chemokine ligand (CCL), CHEMERIN, and growth arrest‐specific (GAS) and they were further confirmed by a chord diagram, respectively (Figure 6E–I). Through stratigraphy of these chord maps, we identified ICAM (Figure S6C), JAM (Figure S6D), CCL (Figure S6E), CHEMERIN (Figure S6F), and GAS (Figure S6G) pathways with relatively contributing ligands in aHSCs and receptors in neutrophils, Kupffer cells, and B cells in the control, CCl_4_, and MenSC groups (Table S5). The aHSCs expressed ligands in five signaling pathways in the control, CCl_4_ and MenSC groups (Figure 6J). The expression of receptors in the five signaling pathways was studied in neutrophils (Figure 6K), Kupffer cells (Figure 6L), and B cells (Figure 6M), respectively. The results showed that the ICAM signaling pathway may be regulated by a combination of the ligand *Icam1* in aHSCs (Figure 6J, blue box markup) and its receptors *Itgal*, *Itgam*, and *Itgb2* in neutrophils (Figure 6K, blue box markup). In the aHSCs population, the expression of *Icam1* was significantly higher in the CCl_4_ group than in the control and MenSCs groups (Figure 6N). Similarly, in neutrophils, the expression of *Itgal* (Figure 6O), *Itgam* (Figure 6P), and *Itgb2* (Figure 6Q) was significantly higher in the CCl_4_ group than in the control and MenSCs groups. Furthermore, a whole‐group expression spectrum analysis revealed that *Icam1* was highly expressed in fibroblasts/HSCs, and *Itgal*, *Itgam*, and *Itgb2* were highly expressed in neutrophils (Figure 6R).

### 2.7 Potential genes in subpopulations of neutrophils for ameliorating liver fibrosis, as shown by scRNA‐seq

**FIGURE 7:**
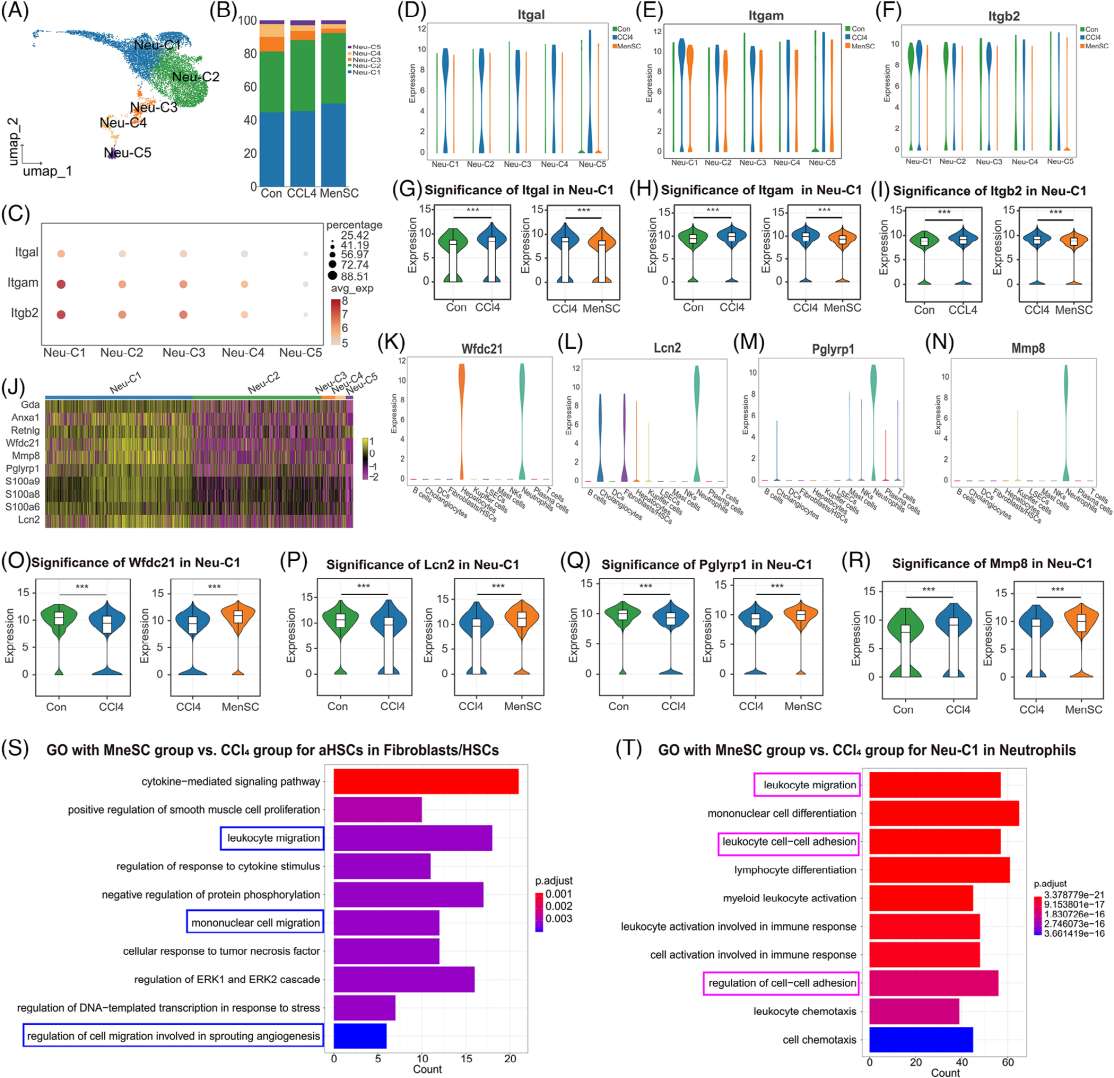
Potential vital genes in subpopulation of neutrophils were revealed for ameliorating liver fibrosis by the scRNA‐seq. (A) Neutrophils were reclustered for five subgroups named as Neu‐C (neutrophils cluster)‐1, Neu‐C2, Neu‐C3, Neu‐C4, and Neu‐C5. (B) The relative proportion of Neu‐C1–C5 in the Con group, CCl_4_ group, and MenSC group. (C) A dotplot for the expression of *Itgal*, *Itgam*, and *Itgb2* in the Neu‐C1–C5. The expression of *Itgal* (D), *Itgam* (E), and *Itgb2* (F) in Neu‐C1–C5 in the Con group, CCl_4_ group, and MenSC group. The *Itgal*, *Itgam*, and *Itgb2* were the most strongly expressed in the Neu‐C1. The expression of *Itgal* (G), *Itgam* (H), and *Itgb2* (I) was significantly higher in the CCl_4_ group than in the Con group, and decreased after MenSC transplantation by two‐by‐two differential significance expression comparison analysis. (J) Top 10 molecules with higher expression in Neu‐C1 compared with other subpopulations. The expression of *Wfdc21* (K), *Lcn2* (L), *Pglyrp1* (M), and *Mmp8* (N) in 12 main cell types in liver cells. The expression of *Wfdc21* (O), *Lcn2* (P), *Pglyrp1* (Q), and *Mmp8* (R) in Neu‐C1 by two‐by‐two differential expression comparison analysis. (S) GO analysis of the subpopulation of aHSCs in the MenSC group and the CCl_4_ group, and cell migration acted as the major mode of action (blue box labeling). (T) GO analysis of the Neu‐C1 in MenSC group and CCl_4_ group, and cell migration and cell adhesion acted as the main mode of action (labeled with pink boxes).

The neutrophils were reclustered into five subgroups: Neu‐C (neutrophil cluster)‐1, Neu‐C2, Neu‐C3, Neu‐C4, and Neu‐C5 (Figure 7A). The heatmap with top‐5 markers of DEGs in five subgroups of neutrophils was presented (Figure S7A). A cell percentage analysis revealed that the highest percentage of neutrophils was found in Neu‐C1 and Neu‐C2 (Figure 7B). A dotplot showed that *Itgal*, *Itgam*, and *Itgb2* were most highly expressed in the Neu‐C1 compared with those in Neu‐C2–C5 (Figure 7C). We split Neu‐C1–C5 in which *Itgal* (Figure 7D), *Itgam* (Figure 7E), and *Itgb2* (Figure 7F) were expressed in the control, CCl_4_, and MenSCs groups. They were not only most strongly expressed in Neu‐C1 (Figure 7C) but were also expressed at higher levels in the CCl_4_ group than in the control group and tended to decrease after the MenSC treatment. A two‐by‐two differential significance expression comparison analysis showed that the expression of *Itgal* was significantly higher in the CCl_4_ group than in the control group, and it was significantly decreased after the MenSC treatment (Figure 7G). The expression trends of *Itgam* (Figure 7H) and *Itgb2* (Figure 7I) were consistent with those of *Itgal*. These results imply that Neu‐C1 is the major effector subpopulation of *Icam1* in aHSCs ligand and *Itgal/Itgb2* and *Itgam/Itgb2* act as receptors for subsequent signal transduction.

To explore the specific expression properties of Neu‐C1, the top‐10 genes with higher expression in Neu‐C1 compared with that in other subpopulations were assessed (Figure 7J). The pseudotime analysis found that Neu‐C1 was mainly distributed in stages 2 and 3 (Figure S7B,C). Additionally, 8 of the 10 most expressed genes, including *Anxa1, Retnlg, Wfdc21, Mmp8*, peptidoglycan recognition protein 1 (*Pglyrp1*)*, S100a8, S100a9*, and lipocalin‐2 (*Lcn2*), clustered together (Figure S7D). Of these molecules, *S100a8, S100a9*, and *Retnlg* are well known as canonical neutrophil markers,^55^ and the relative expression profiles of the three genes (Figure S7E–G) were further verified in the present study. *Wfdc21* was expressed only in hepatocytes and neutrophils (Figure 7K), suggesting that neutrophil–hepatocyte crosstalk may occur through this factor. *Lcn2* was mainly expressed in neutrophils, but cholangiocytes and fibroblasts/HSCs also expressed *Lcn2* (Figure 7L). Further studies revealed *Anxa1* (Figure S7H) to be nonspecific, whereas *Pglyrp1* (Figure 7M) and *Mmp8* (Figure 7N) were almost exclusively expressed in neutrophils and could be considered as genes specifically expressed in neutrophils. A two‐by‐two differential significance expression comparison analysis revealed that the expression of *Wfdc21* was significantly lower in the CCl_4_ group than in the control group and significantly increased after the MenSC treatment (Figure 7O). The expression trends of *Lcn2* (Figure 7P) and *Pglyrp1* (Figure 7Q) were consistent with those of *Wfdc21*. These results indicate that *Wfdc21, Lcn2*, and *Pglyrp1* in Neu‐C1 can serve as potential targets or effector molecules with a negative correlation trend. In the Neu‐C1 cell population, the expression of *Mmp8* was significantly higher in the CCl_4_ group than in the control group and remained significantly elevated after the MenSC injection (Figure 7R).

A GO analysis of the subpopulation of aHSCs in the MenSC and CCl_4_ groups (Figure 7S) revealed that cell migration was the major mode of action (blue boxes). In addition, a GO analysis of the Neu‐C1 subpopulation in the MenSC and CCl_4_ groups (Figure 7T) revealed that cell migration and adhesion were the main modes of action (pink boxes). These results suggested aHSC and Neu‐C1 showed strong differences in cell migration and adhesion between the MenSC and CCl_4_ groups.

### 2.8 Colocalization for ICAM signaling interaction in HSCs and neutrophils by the immunofluorescence

**FIGURE 8:**
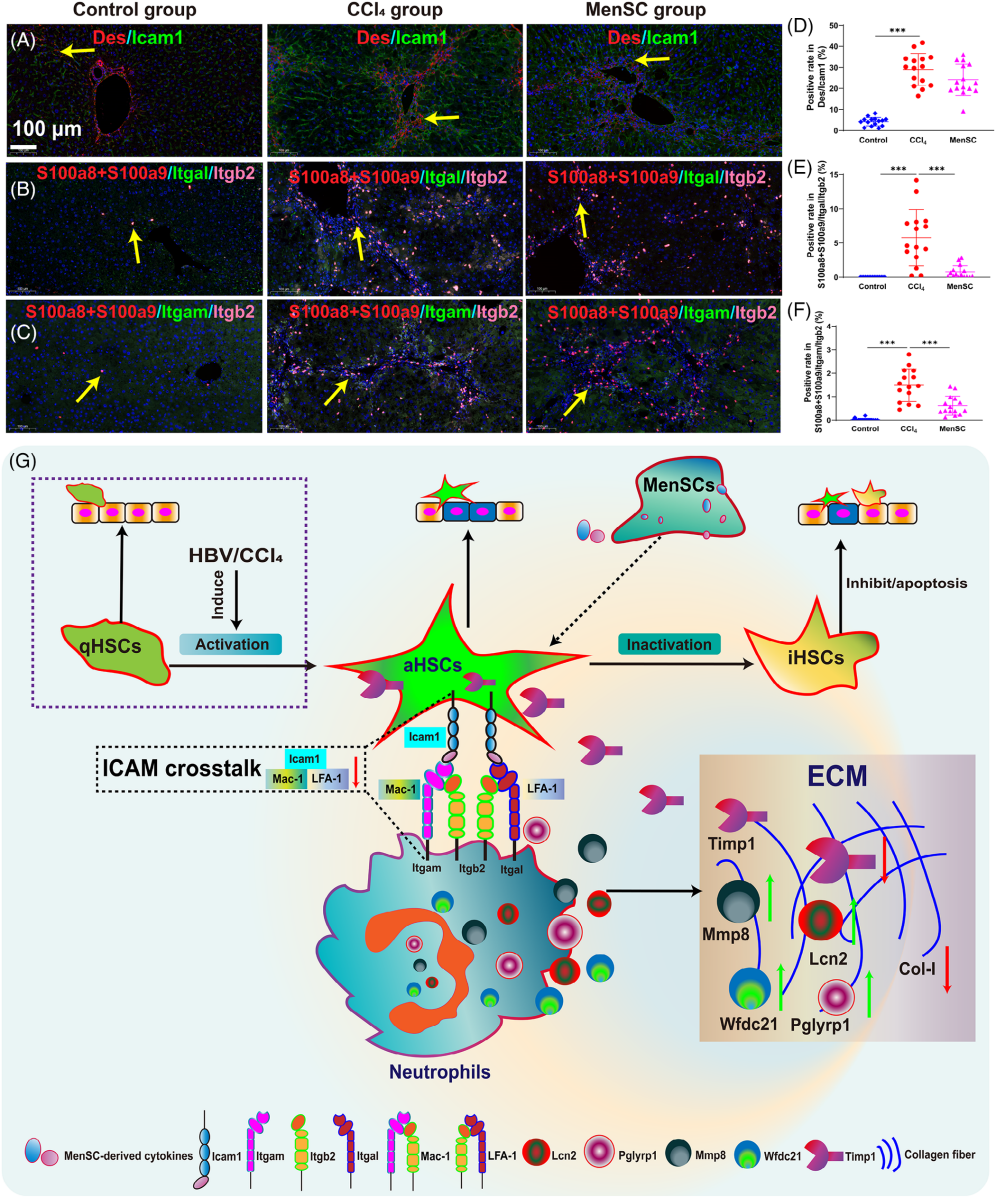
Colocalization for intercellular adhesion molecule (ICAM) signaling interaction in HSCs and neutrophils by the immunofluorescence. (A) *Icam1* expression was checked on HSCs (marked as *Des*) using immunofluorescence. (B) LFA‐1 (*Itgal*/*Itgb2*) expression on neutrophils (*S100a8*+*S100a9*) using immunofluorescence. (C) Mac‐1 (*Itgam*/*Itgb2*) expression on neutrophils (*S100a8*+*S100a9*) using immunofluorescence. The positive rate of *Des/Icam1* (D), *S100a8+S100a9/Itgal/Itgb2* (E), *S100a8+S100a9/Itgam/Itgb2* (F) in the control group, CCl_4_ group, and MenSC group. Three samples were used for each group and they were randomly captured with five horizons. (G) A specific mechanistic diagram for MenSC in the treatment of liver fibrosis in HBV‐Tg mice. HBV nucleic acid analogue is the sufficient stimulant for inducing qHSCs into aHSCs. Reducing the intercellular adhesion of ICAM signaling crosstalk between aHSCs and neutrophils is one of the underlying mechanisms. For detail, MenSC secreted cytokines acted on aHSC to reduce ligand of Icam1 in aHSC, and Icam1 specifically binds LFA‐1 (a complex of *Itgal/Itgb2*) and Mac‐1 (a complex of *Itgam/Itgb2*) in neutrophils, resulting in decreasing adhesion between aHSCs and neutrophils. *Lcn2*, *Pglyrp1*, *Wfdc21*, and *Mmp8* may be used as potential and novel targets in neutrophils for decreasing liver fibrosis. Yellow arrow represents the positive site. Asterisks (*) marked the significant differences (***p < 0.001). Error bars indicated by SD.

By focusing on ICAM signaling in HSCs and neutrophils, the *Icam1* expression was checked on HSCs (marked as *Des*) using immunofluorescence (Figure 8A). And LFA‐1 (*Itgal/Itgb2*) expression on neutrophils (*S100a8*+*S100a9*) using immunofluorescence (Figure 8B). Mac‐1 (*Itgam/Itgb2*) expression on neutrophils (*S100a8*+*S100a9*) using immunofluorescence (Figure 8C). Although the positive rate of *Des*/*Icam1* was reduced by immunofluorescence (Figure 8D, average 28.99% in CCl_4_ group and average 24.13% in MenSC group), however, there are no statistical discrepancy. The coexpression of *S100a8+S100a9/Itgal/Itgb2* (Figure 8E) and *S100a8*+*S100a9*/*Itgam/Itgb2* (Figure 8F) were almost 0 in the Control group. The positive rate of *S100a8+S100a9/Itgal/Itgb2* in MenSC group (average 0.74%) were significantly decreased in contrast to the CCl_4_ group (average 5.78%) (Figure 8E). Similarly, the positive rate of *S100a8+S100a9/Itgam/Itgb2* in MenSC group (average 0.62%) were significantly decreased in contrast to the CCl_4_ group (average 1.49%) (Figure 8F). MenSCs secrete cytokines that act on aHSCs to reduce the expression of the aHSC ligand, *Icam1*, which specifically binds to LFA‐1 (*Itgal/Itgb2*) and Mac‐1 (*Itgam/Itgb2*) in neutrophils. Furthermore, the mechanistic diagram is shown (Figure 8G).

## 3 DISCUSSION

HSCs have been implicated as the most critical cell population in the progression of liver fibrosis.^15^, ^21^, ^56^, ^57^ Several studies have indicated that MSCs can improve liver fibrosis through paracrine effects, mainly by targeting HSCs.^58^, ^59^ Shi et al.^60^ reported that the transplant of umbilical cord derived MSCs can significantly improve the long‐term survival rate and liver function of patients with HBV‐related decompensated liver cirrhosis. Compared with MSCs derived from other tissue sources, MenSCs possess certain unique advantages, including abundant yield, convenient material collection, periodic acquisition, strong proliferation, and low immune rejection, and there are few ethical disputes regarding their use.^61^, ^62^, ^63^ First, the collection of MenSCs is convenient because menstrual blood is a renewable biological resource, which relatively simplifies the acquisition of MenSCs and does not cause physical harm or discomfort to the donor. In contrast, the extraction of BM‐MSCs usually requires bone marrow aspiration, which not only causes the patient some pain but also poses certain risks and complications. Second, MenSCs have a strong proliferative capacity. Research has shown that MenSCs exhibit a rapid proliferation rate and cell doubling time when cultured in vitro, making them potentially valuable in tissue engineering and regenerative medicine.^64^ In addition, no ethical controversies are associated with the procurement of MenSCs. Particularly, previous studies have confirmed that MenSCs treat severe COVID‐19 in clinical trials.^65^, ^66^ Therefore, MenSCs are gradually becoming a focal point in the field of stem cell scientific research and clinical application owing to their convenient collection, periodic availability, excellent proliferative capacity, and the absence of ethical issues, among other advantages. In this study, we provide preliminary data using MenSCs for the treatment of HBV‐associated liver fibrosis in HBV‐Tg mice.

HBsAg levels were continuously decreased after MenSC transplantation. The possible reason is that MenSCs regulate immunity via the immune cells (such as macrophages/Kupffer cells) in the mouse body. MenSCs increase the phagocytic function of macrophages by changing their polarity, which accelerates the clearance of HBV‐related indicators without entering the peripheral blood.^67^ As a result, these virological indicators in the peripheral blood have decreased. Anyhow, the specific mechanism requires further research. Under physiological conditions, qHSCs reside in the Diss space and exhibit a quiescent phenotype expressing vitamin A, nerve growth factor p75, *Lrat*, *Gfap*, and *Pparγ*.^68^, ^69^ We found no qHSCs in any of the groups according to scRNA‐seq; therefore, the activation of HSCs is a complete event in HBV‐Tg mice. HBV‐Tg mice produce inflammation but not fibrosis.^48^ The HBV nucleic acid analog is the stimulant for inducing qHSCs into aHSCs. This suggests that the proportion and number of HSCs may be different in HBV‐Tg mice than in C57BL/6 mice with CCl_4_‐induced liver fibrosis. In the present study, the proportion of fibroblasts/HSCs and aHSCs subpopulations was higher in the control group than in the CCl_4_ and MenSC groups, which is contrary to that in most studies.^25^, ^29^ A possible explanation is that the different markers cannot reach a consensus, especially the reclustering of the fibroblast/HSC subpopulation. Currently, we lack a uniform and perfect nomenclature system for defining the cell types of fibroblasts/HSCs. Some of the variations detected in isolated cells by scRNA‐seq do not reflect in vivo physiology owing to the heterogeneity.^70^ We have checked the database and identified certain double‐positive cells for the subpopulation of fibroblasts/HSCs, although the doublets have been effectively filtered out. Yang et al.^71^ found dual‐featured cell type of immune‐featured decidual stromal cells using scRNA‐seq. Recently, Domcke and Shendure^72^ advocated a data‐driven tree‐based nomenclature capable of spanning species‐specific life cycles. It is expected to establish a taxonomic classification with a reference cell tree similar to species naming and develop a more stable and scalable precise scientific communication framework.^72^ Additionally, the loss of qHSCs in HBV‐Tg mice may be a pivotal event in regulating the ratio of fibroblast/HSC subpopulations. To confirm this assumption, HBV‐Tg mice in the embryo and adult stages are needed to check the expression of qHSCs (such as *Lrat, Reln*, and *Gfap*) and the proportion of qHSCs/aHSCs in the future.

HSC inactivation also exists in other CLDs, including NASH; therefore, we looked for such commonalities.^73^ Rosenthal et al.^33^ found that NASH livers have four different HSC clusters, one of which is the cluster of iHSCs after the rejuvenation of NASH, similar to qHSCs; however, their expression profiles were quite different between iHSCs and qHSCs, and iHSCs were closely related to the inflammatory type. However, iHSCs express aHSC markers, making it difficult to distinguish between iHSCs and aHSCs. Fortunately, aHSCs cannot express or lowly express the unique genes of *Tcf21, Smoc2*, and *Fbln7* in iHSCs, which have been verified by other studies.^33^, ^34^ Except for some classic markers, these genes (such as *Dpt*, *Gsn*, and *Saa3*) may be key for the transformation of aHSCs into iHSCs during liver fibrosis.

Using scRNA‐seq, we identified the underlying mechanism of MenSCs in the treatment of liver fibrosis in HBV‐Tg mice, which was the reduction of ICAM signaling between aHSCs and neutrophils. Integrin‐dependent microtubule stabilization contributes to cell adhesion during cell migration through ECM sensing.^74^ Recently, Wei et al.^75^ found that β1 integrin (*Itgb1*) is the key and conserved molecule for cell surface interactions and transitional information in regulating ECM signaling between embryonic and extraembryonic lineages with stem cell coculture. Additionally, the blockade of *Icam1* lowered the expression of fibrosis genes, decreased hepatic neutrophil infiltration, and mitigated liver fibrosis.^35^ The overexpression of *Itgam* resulted in the deterioration of liver function during liver cirrhosis.^76^ In the present study, MenSCs secrete cytokines that act on aHSCs to reduce the expression of the aHSC ligand, ICAM1, which specifically binds to Mac‐1 and LFA‐1 in neutrophils. The binding of these two molecules was also attenuated, as evidenced by a decrease in the expression of *Itgam, Itgal*, and *Itgb*2. This induced the action of many intracellular molecules. Of these, *Mmp8* inhibits *Timp1*, and *Wfdc21* is a collagen‐degrading enzyme that degrades collagen fibers. *Pglyrp1* and *Lcn2* have immunomodulatory effects. Because *S100a8, S100a9*, and *Retnlg* are recognized neutrophil markers, the functions of these factors were not examined in the present study. In conclusion, *Wfdc21*, *Lcn2, Pglyrp1*, and *Mmp8* may be potential and novel neutrophil targets to decrease liver fibrosis.

In addition to the interactions among aHSCs, neutrophils, and the ICAM pathway, other potential mechanisms need to be investigated. The current study had some limitations. Because transplanted human MenSCs are difficult to identify in vivo owing to the lack of distinctive markers to check the few MenSCs, even when scRNA‐seq technology is used, other novel methods are needed to accurately capture human cells when they are transplanted into mice. Moreover, HBV is a hepatotropic virus with strict specificity for humans and chimpanzees.^77^ Thus, an HBV‐related liver fibrosis disease mouse model is immensely lacking, and cell classification, especially for HSCs/fibroblasts, is not yet ideal. The relative numbers of aHSCs/iHSCs were also small, which means that contingency may have affected the scRNA‐seq database. Additionally, Monocle2 was used for the pseudotime. CytoTRACE is a novel technology for further research the developmental potential.^78^ Further studies are needed to fully understand the functional contribution of specific alterations in cell subsets or other potential signaling pathways during liver fibrosis and to translate these findings into effective therapeutics with some novel technologies, such as generating human liver organoids in disease model.^79^ Additionally, more experiments should be explored in vitro with different primary cells or cell lines.

In summary, MenSCs exert therapeutic effects in composite HBV‐Tg mouse liver fibrosis model. The fibroblast/HSC subpopulation investigated herein was reclustered using scRNA‐seq and revealed the heterogeneity of fibroblasts/HSCs. We uncovered the cell–cell adhesion interacting between aHSCs and neutrophils and highlighted the underlying molecular mechanisms via ICAM crosstalk.

## 4 MATERIALS AND METHODS

### 4.1 HBV transgenic mice

Male HBV‐Tg mice (GenBank No. AF305422.1) aged 7−8 weeks were purchased from Beijing Vitalstar Biotechnology Co., Ltd. (Beijing, China).^80^ The experimental mice, excrements, litter, and related consumables were processed in accordance with the requirements of ABSL‐2 (BSL‐2). The animal experiments were approved jointly by the Animal Care and Use Committee of the First Affiliated Hospital, Zhejiang University School of Medicine (No. 2022‐1082) and the Beijing Vitalstar Biotechnology (No. VST‐SY‐20221018).

### 4.2 Source and preparation of MenSCs

Human MenSCs (no. 00612−210415P), provided by the Innovative Precision Medicine (IPM) Group (Hangzhou, China), were acquired from a healthy female donor who provided informed consent before donation, as described in previous studies.^47^, ^65^, ^81^ In brief, menstrual blood samples were collected and cultured in α‐MEM (Invitrogen, CA, USA) culture media supplemented with 15% fetal bovine serum (Gibco, Australia). After reaching 80−90% confluence, MenSCs were washed, harvested, resuspended, and cryopreserved. To identify MenSC surface molecular markers, fifth‐generation MenSCs were digested with trypsin‐EDTA (Gibco). The cells were then precipitated and resuspended by adding an appropriate amount of Stain Buffer (BD Biosciences, USA). CD29, CD34, CD45, CD73, CD90, CD105, CD117, and HLA‐DR were added to the Eppendorf tubes, according to the dosage recommended by the antibody manufacturers. The detail procedure has been previously described in our previous studies.^47^, ^81^ IgG1 or IgG2a served as isotype controls; detailed information is presented in Table S6. To identify the three‐line differentiation of MenSCs, we used the OriCell^®^ human bone marrow MSC osteogenic, adipogenic, and chondrogenic induction differentiation kit (Cyagen Biosciences, Guangzhou, China) according to the instructions. The detail three‐line differentiation procedure has been previously described.^81^, ^82^ After approximately four weeks, the MenSCs differentiated into osteoblasts, adipocytes, and chondrocytes, after which they were stained and photographed.

### 4.3 CCl_4_‐induced liver fibrosis and MenSC transplantation

HBV‐Tg mice do not spontaneously develop liver fibrosis. The control group (*N* = 8) was injected with corn oil (Macklin Biochemical Technology Co., Ltd., Shanghai, China). To induce liver fibrosis in the mice, the experimental groups were intraperitoneally injected with CCl_4_ (Macklin Biochemical Technology Co., Ltd.; dissolved in corn oil at 20% solution, 2 mL/kg body weight) twice weekly for 6 weeks. MenSCs were administered in different doses (MenSC‐low group, 1 × 10^7^ cells/kg, *N* = 8; MenSC‐high group, 4 × 10^7^ cells/kg, *N* = 8) in week 7, and an equal amount of solution (CCl_4_ model group, *N* = 8) was used for the control. Serological indicators were detected at the time point after 1 week of MenSC administration (MenSC 1 W), and all samples were collected at the time point of MenSC 2 W (Figure 1A).

### 4.4 Cell tracking in CCl_4_‐induced liver fibrosis

For cell tracking experiments in vivo, DiR (DiIC18(7), Thermo Fisher) was used to label the MenSCs in CCl_4_‐induced liver fibrosis HBV‐Tg mice. Follow the kit instructions, DiR was diluted to the final concentration of 10 µg/mL. MenSCs were diluted to the concentration of 5 × 10^6^ cells/mL. DiR‐labeled MenSCs (1 × 10^6^ cells, 200 µL) were then injected into the mouse spleens, with 10 mice in the group being treated. An IVIS small animal optical imaging system (IVIS Lumia series III; PerkinElmer, MA, USA) was used to detect the distribution and intensity of the fluorescence signals before the MenSC injections and 16 h, 24 h, 3 days, 5 days, 7 days, 11 days, and 14 days after the MenSC injections (Figure 1A). On days 3 and 14 after the DiR‐MenSC transplantation, the livers, lungs, spleens, kidneys, and hearts were removed for tissue photography.

### 4.5 Serum liver function and qRT‐PCR

The mice were weighed once a week (D1‐D56). The sera were sent for liver function testing by the Dian Diagnostics Medical Laboratory (Beijing, China), including testing for the liver function indices ALT, AST, ALP, and ALB and serum HBV‐related virological indicators, including HBV DNA, HBsAg, and HBeAg.

Total RNA from the mouse livers was extracted by the manufacturer's instructions for the TRIzol reagent (Invitrogen, MA, USA), using TransScript® One‐Step gDNA Removal and cDNA Synthesis SuperMix (TransGen Biotech, Beijing, China). A qRT‐PCR analysis was performed using UltraSYBR Mixture (CoWin Biosciences, Beijing, China) with the Applied Biosystems 7500. The detection indicators were HBV product (X‐universal), TGF‐β1 (*Tgfb1*), α‐SMA (*Acta2*), and Col‐I (*Col1a1*). GAPDH was used as an internal control. The primer sequences are presented in Table S7.

For coculturing experiment in vitro, recombinant human TGF‐β1 (7745‐BH; R&D System, USA) stimulated LX‐2 cells (human HSC cell line; Wuhan Procell Life Science & Technology Co., Ltd, China) for 24 h to activate HSCs. MenSC and LX‐2 were cocultured in six‐well transwell (Corning, USA) for three biological duplication. Individual LX‐2 cells named as LX‐2 group, MenSC and LX‐2 in the transwell named as coculture group. The *Icam1* expression of LX‐2 cells in LX‐2 group and coculture group were assessed by qRT‐PCR. The β‐actin was used as an internal control. The primer sequences are presented in Table S7.

### 4.6 Histological staining, IHC, and immunofluorescence

Histopathological indicators were assessed at the experimental endpoint (D56/MenSC 2 W). HE staining and SR staining were performed after routine paraffin section preparation (Jkgreen Technology, Beijing, China). The histological characteristics of the liver lesions were used to evaluate the degrees of the liver lesions. NASH activity and liver fibrosis scores were evaluated using the SAF scoring system.^49^ IHC was used to identify α‐SMA, Col‐I, and TGF‐β1, using a Dako EnVision IHC system (Dako, Denmark) as previously described.^47^, ^82^ Immunofluorescence was performed to examine *Des*, *Icam1*, *S100a8*+*S100a9*, *Itgal*, *Itgam*, and *Itgb2* after paraffin section preparation with the instruction by HaoKe Biotechnology Co. Ltd., Hangzhou, China. DAPI (HKI0005; HaoKe Biotechnology) was used for staining nucleus, and the coexpression of *Des/Icam1*; *S100a8+S100a9/Itgal/Itgb2*; and *S100a8+S100a9/Itgam/Itgb2* were statistically analyzed in control group, CCl_4_ group, and MenSC group. Three independent samples were used for each group with five horizons. DAB solution (HaoKe Biotechnology) was used for color development, the duration of which was controlled under a microscope (NIKON‐ECLIPSE C1). The detailed antibody information and usage are shown in Table S6.

### 4.7 RNA‐bulk analysis for HBV‐Tg mouse livers

For mRNA deep (or RNA‐bulk) sequencing, RNA samples were prepared using TruSeq RNA Sample Preparation and performed by Beijing CapitalBio Technology. In brief, mRNA molecules containing poly‐A were purified from total RNA using poly‐T oligonucleotide‐conjugated magnetic beads. The cDNA fragments were purified and the “A” tails were ligated. The cDNA library was constructed (identified by Agilent 2100 Bioanalyzer). The resulting libraries were sequenced on the NovaSeq 6000 platform according to the confidence intervals used for the serial analysis of gene expression data analysis.^83^

### 4.8 Single‐cell capture and cDNA synthesis for mouse livers

A mouse liver dissociation kit (Miltenyi Biotec, Germany) was used to digest the livers by gentle shaking at 37°C for 30 min. Filtration was performed through a Falcon^®^ 70‐µM cell filter (BD Biosciences), and the samples were centrifuged at 300 *g* for 6 min to collect single‐cell suspensions. To comprehensively define HBV‐Tg mouse livers at the single‐cell level, we collected HBV‐Tg mouse liver NPCs and partial liver parenchymal cells (hepatocytes/cholangiocytes) from the normal control (Con group), CCl_4_ fibrosis model (CCl_4_ group), and MenSC high‐dose therapy (named as MenSC group) at the time point of MenSC 2 W (Figure 1A). Liver NPCs and partial liver parenchymal cells from three groups of nine HBV‐Tg mice (three samples per group) were subjected to scRNA‐seq using the 10× Genomics platform provided by Beijing CapitalBio Technology (Beijing, China). The scRNA‐seq was performed using the Single‐Cell 3′ Library and Gel Bead Kit V3.1 (10× Genomics, 1000121) and Chromium Single‐Cell G Chip Kit (10× Genomics, 1000120) for cell capture and cDNA synthesis. Cell suspensions were loaded onto the Chromium Single Cell Controller (10× Genomics) to generate single‐cell gel beads, according to the manufacturer's instructions. In brief, single cells were suspended in PBS containing 0.04% bovine serum albumin (Sigma, USA). RNA was barcoded by the reverse transcription of an individual GEM in turn released. Reverse transcription was performed using a S1000TM Touch Thermal Cycler (Bio‐Rad, CA, USA). The cDNA was amplified, and its quality was evaluated for subsequent analysis using an Agilent 4200 (CapitalBio Technology).

### 4.9 ScRNA‐seq library preparation and data processing

ScRNA‐seq libraries were established using Single‐Cell 3′ Libraries and Gel Bead kits according to the manufacturer's protocols. Sequencing was performed using an Illumina NovaSeq6000 sequencer at a sequencing depth over 100,000 reads per cell (CapitalBio Technology). Barcode and UMI counting were performed using the CellRanger counting module to generate a feature barcode matrix and identify clusters. Dimensionality reduction was performed using a principal component analysis (PCA) to generate clusters with K‐means and graph‐based algorithms. Another clustering method used was Seurat 3.0 (R package), where cells with gene counts less than 200 or gene counts ranked in the top 1% were considered abnormal and filtered out. During the analysis or preparation of the cell samples, cells with a mitochondrial gene content exceeding 25% were removed from the dataset. Dimensionality reduction was performed using a PCA, and visualization was achieved by t‐SNE and UMAP.^84^ All the genes in the cells of a sample were extracted using gene set enrichment analysis software. The cells were quasi‐temporally sorted using DEGs identified with Seurat. The merging of multiple samples was based on the Harmony algorithm in Seurat.^85^ The analyzed single‐cell trajectory obtained with the “plot_cell_trajectory” function was constructed using Monocle2 (R package) with the introduction of pseudotimes.

### 4.10 Enrichment and CellChat analysis

GO enrichment terms and KEGG enrichment pathways were functionally analyzed, classified, and annotated using the ClusterProfiler package in R.^86^ Fisher's exact test was used to calculate the differences in representative GO function sets. A KEGG functional analysis was performed to annotate and categorize the pathways.^87^ To predict cell–cell communication between different cell types, we applied the CellChat R package to the scRNA‐seq data. The quantification of signaling pathways is derived from important ligand–receptor pairs between cell types. In the computeCommunProbe function, CellChat determined the effects of the proportion of cells in each cell group on the probability calculations. This was accomplished by separately applying the dot heatmap and chord plots using the netVisual_bubble and netVisual_chord_gene functions.

### 4.11 Statistical analysis

Statistical analyses were performed using GraphPad Prism 9 software. The data were exhibited as the mean ± standard deviation and analyzed using Student's *t*‐tests, *χ* ^2^ tests, or a one‐way analysis of variance. Correlation analyses were performed using Spearman's rank‐order correlations. Statistical significance was set at *p < 0.05, **p < 0.01, and ***p < 0.001.

## AUTHOR CONTRIBUTIONS

Charlie Xiang and Lanjuan Li conceived and designed this study. Lijun Chen, Yuqi Huang, Ning Zhang, Jingjing Qu, Yangxin Fang, Jiamin Fu, Yin Yuan, Qi Zhang, and Zuoshi Wen performed the experiments, collected and analyzed the data, and wrote the manuscript. Hang Li, Li Yuan, Lu Chen, Zhenyu Xu, Yifei Li, Huadong Yan, and Hiromi Izawa collected and analyzed the data, and revised the manuscript. All authors have read and approved the final manuscript.

## CONFLICT OF INTEREST STATEMENT

Hang Li, Li Yuan, Lu Chen, and Zhenyu Xu are employees of the Innovative Precision Medicine (IPM) Group. The other authors declare that they have no competing interests.

## ETHICS STATEMENT

The animal experiments were approved jointly by the Animal Care and Use Committee of the First Affiliated Hospital, Zhejiang University School of Medicine (No. 2022‐1082) and the Beijing Vitalstar Biotechnology (No. VST‐SY‐20221018). Human MenSCs were provided by the Innovative Precision Medicine (IPM) Group. Written informed consent was obtained from the donor.

## Supporting information

Supporting Information

## Data Availability

The raw data of single‐cell RNA sequencing (No. CRA014464) and RNA‐bulk sequencing (No. CRA016016) have been deposited in the Genome Sequence Archive. The detail datasets and materials are available from the corresponding authors upon reasonable request.
