# Supplementary material for: Single‐cell RNA sequencing reveals reduced intercellular adhesion molecule crosstalk between activated hepatic stellate cells and neutrophils alleviating liver fibrosis in hepatitis B virus transgenic mice post menstrual blood‐derived mesenchymal stem cell transplantation
Source: MedComm (2020). 2024 Jul 22;5(8):e654. doi: 10.1002/mco2.654 (PMC11261812; doi:10.1002/mco2.654)
Supplement: Supplementary file 1 — Supporting Information [file MCO2-5-e654-s001.docx]

***Supplementary Materials***

**Single-cell RNA sequencing reveals reduced intercellular adhesion molecule** **crosstalk between** **[activated](javascript:;) hepatic stellate cells and** **neu****trophils alleviating liver fibrosis in** **hepatitis B virus transgenic mice post** **menstrual blood-derived mesenchymal stem cell transplantation**

Lijun Chen^1^^, 2^, Yuqi Huang^1^, Ning Zhang^1^, Jingjing Qu^3^, Yangxin Fang^1^, Jiamin Fu^1^, Yin Yuan^1^, Qi Zhang^1^, Hang Li^4^, Zuoshi Wen^5^, Li Yuan^4^, Lu Chen^4^, Zhenyu Xu^4^, Yifei Li^1, 2^, Huadong Yan^6^, Hiromi Izawa^7^, Lanjuan Li^1, 2, 8^*, Charlie Xiang^1, 2, 8^*

1. State Key Laboratory for Diagnosis and Treatment of Infectious Diseases, National Clinical Research Center for Infectious Diseases, National Medical Center for Infectious Diseases, Collaborative Innovation Center for Diagnosis and Treatment of Infectious Diseases, The First Affiliated Hospital, Zhejiang University School of Medicine, Hangzhou, Zhejiang, 310003, China

2. Research Units of Infectious Disease and Microecology, Chinese Academy of Medical Sciences, Beijing, 100730, China

3. Department of Respiratory Disease, Thoracic Disease Centre, The First Affiliated Hospital, Zhejiang University School of Medicine, Hangzhou, Zhejiang, 310003, China

4. Innovative Precision Medicine (IPM) Group, Hangzhou, Zhejiang, 311215, China

5. Department of Cardiology, The First Affiliated Hospital, Zhejiang University School of Medicine, Hangzhou, Zhejiang, 310003, China

6. Infectious disease department, Shulan (Hangzhou) Hospital Affiliated to Zhejiang Shuren University Shulan International Medical College, Hangzhou, Zhejiang, 310022, China

7. Jingugaien Woman Life Clinic, Jingu-Gaien 3-39-5 2F, Shibuya-Ku, Tokyo, Japan

8. Jinan Microecological Biomedicine Shandong Laboratory, Jinan, Shandong, 250117, China

* Correspondences: **Prof. Charlie Xiang**, [cxiang@zju.edu.cn](mailto:cxiang@zju.edu.cn); **Prof. Lanjuan Li**, [ljli@zju.edu.cn](mailto:ljli@zju.edu.cn).

**Table S1**. Observing the Hematoxylin and eosin (HE) and Sirius red (SR) staining photos of tissue samples, the scores of 32 HBV-Tg mice slice specimens.

| **Groups** | **No.** | **Animal No.** | **Scores (HE + SR)** | | | | | | | **Other usage** |
| --- | --- | --- | --- | --- | --- | --- | --- | --- | --- | --- |
|  |  |  | I(0-2) | S(0-3) | G(0-3) | B(0-2) | D(0-2) | F(0-4) | N(0-3) |  |
| **Normal control** | 1 | 01 | 0 | 0 | 0 | 1 | 0 | 0 | 0 |  |
|  | 2 | **02** | 0 | 0 | 0 | 1 | 0 | 0 | 0 | ScRNA-seq |
|  | 3 | **03** | 0 | 0 | 0 | 1 | 0 | 0 | 0 | ScRNA-seq |
|  | 4 | 04 | 0 | 0 | 0 | 1 | 0 | 0 | 0 |  |
|  | 5 | **05** | 0 | 0 | 0 | 1 | 0 | 0 | 0 | ScRNA-seq |
|  | 6 | 53 | 0 | 0 | 0 | 1 | 0 | 0 | 0 |  |
|  | 7 | 54 | 0 | 0 | 0 | 1 | 0 | 0 | 0 |  |
|  | 8 | 55 | 0 | 0 | 0 | 1 | 0 | 0 | 0 |  |
| **CCl_4_ model** | 1 | 17 | 2 | 0 | 3 | 2 | 1 | 3 | 1 |  |
|  | 2 | **37** | 2 | 0 | 3 | 2 | 2 | 4 | 2 | ScRNA-seq |
|  | 3 | **23** | 2 | 0 | 3 | 2 | 1 | 2 | 1 | ScRNA-seq |
|  | 4 | **28** | 2 | 0 | 3 | 2 | 1 | 4 | 2 | ScRNA-seq |
|  | 5 | 59 | 2 | 0 | 2 | 2 | 1 | 3 | 1 |  |
|  | 6 | 60 | 2 | 0 | 3 | 2 | 1 | 3 | 1 |  |
|  | 7 | 61 | 2 | 0 | 3 | 2 | 1 | 3 | 1 |  |
|  | 8 | 63 | 2 | 0 | 3 | 2 | 1 | 3 | 1 |  |
| **MenSC-low** | 1 | 21 | 1 | 0 | 1 | 1 | 1 | 2 | 1 |  |
|  | 2 | 52 | 2 | 0 | 2 | 2 | 2 | 2 | 1 |  |
|  | 3 | 22 | 1 | 0 | 1 | 1 | 0 | 2 | 1 |  |
|  | 4 | 46 | 2 | 0 | 3 | 2 | 2 | 3 | 1 |  |
|  | 5 | 20 | 1 | 0 | 2 | 1 | 1 | 1 | 1 |  |
|  | 6 | 33 | 2 | 0 | 3 | 1 | 1 | 2 | 1 |  |
|  | 7 | 42 | 2 | 0 | 3 | 2 | 2 | 3 | 1 |  |
|  | 8 | 41 | 2 | 0 | 3 | 2 | 1 | 3 | 1 |  |
| **MenSC-high** | 1 | 40 | 1 | 0 | 0 | 1 | 1 | 2 | 1 |  |
|  | 2 | **18** | 2 | 0 | 2 | 1 | 1 | 2 | 1 | ScRNA-seq |
|  | 3 | **51** | 1 | 0 | 2 | 1 | 2 | 2 | 1 | ScRNA-seq |
|  | 4 | 48 | 2 | 0 | 2 | 1 | 1 | 1 | 1 |  |
|  | 5 | 08 | 2 | 0 | 3 | 2 | 2 | 3 | 1 |  |
|  | 6 | **45** | 1 | 0 | 1 | 2 | 0 | 3 | 1 | ScRNA-seq |
|  | 7 | 34 | 2 | 0 | 1 | 1 | 1 | 2 | 1 |  |
|  | 8 | 13 | 2 | 0 | 2 | 2 | 2 | 3 | 1 |  |

**Table S2**. 33 common genes in "CCl_4_ vs. Con up" and "MenSC vs. CCl_4_ down" and 468 common genes in "CCl_4_ vs. Con down" and "MenSC vs. CCl_4_ up".

| **33 common genes in "CCl_4_ vs. Con up" and "MenSC vs. CCl_4_ down"** | | | | | | | | | |
| --- | --- | --- | --- | --- | --- | --- | --- | --- | --- |
| Gm47283 | Angptl7 | Pdcd1 | Trav8n-2 | Ctrb1 | Chil3 | Erdr1 | Trbv3 | Gm6192 | Igkv10-94 |
| Ighv3-1 | Eomes | Igkv4-79 | Tlr9 | Tspan8 | Ankrd1 | Serinc2 | Runx3 | Slc4a11 | Elapor1 |
| Ms4a4c | Igkv3-7 | Spint1 | Itga3 | Reg1 | Sprr1a | Gm44174 | Igkv6-14 | Dsp | Tlr5 |
| Gm18362 | Wnt11 | Ocstamp |  |  |  |  |  |  |  |
| **468 common genes in "CCl_4_ vs. Con down" and "MenSC vs. CCl_4_ up"** | | | | | | | | | |
| Cxcl13 | Cyp26b1 | Marco | Cntfr | Gm7609 | Gm5970 | Cyp4b1 | Gpihbp1 | Ackr4 | Clec14a |
| Tpbgl | Ushbp1 | Exoc3l2 | Arhgef15 | Pcdh12 | Btnl9 | Col13a1 | Fam167b | Erg | Adam23 |
| Tmem204 | Ifi44 | Clec4g | Cd300lg | Cldn5 | Egfl7 | Fam124a | Sox18 | F8 | Oit3 |
| Pla2r1 | Gata4 | Exoc3l | Lyve1 | Ehd3 | Dnase1l3 | Masp1 | Emcn | Mapk12 | Esrrb |
| Ccm2l | Gm4951 | Serpina3f | Gpr182 | Id4 | Ackr2 | Tmem132e | Robo4 | Chst7 | Kdr |
| Plpp1 | Gja4 | Ifi203-ps | Tmcc2 | Asb4 | Ripply3 | Bmp2 | Adgrl4 | Npr1 | Sh3tc2 |
| Prickle1 | Tek | Tspan18 | Dll4 | Myct1 | Ntn4 | Mb21d2 | Fgd5 | Iigp1 | Acer2 |
| Gng11 | Thsd1 | Nr2f1 | Fam171a1 | Alox12 | Wnt2 | Tfpi | Gm10605 | Tmem44 | Afap1l1 |
| Clca3a1 | Enpep | She | Flt4 | Vsig8 | Slc7a2 | Ramp2 | Stab2 | Rasal2 | Ush1g |
| Tbx2 | Sox7 | Plpp3 | Meis2 | Lhx6 | Adm | Clec1b | Nkd1 | Ptch2 | Adgrf5 |
| Ighv1-64 | Rasd1 | Slc26a10 | Hoxa5 | Cyyr1 | Esam | Art3 | Hecw2 | Cavin2 | Cxcl9 |
| Ccdc152 | Ldb2 | Shank3 | Mmrn2 | Tbxa2r | Kcnb1 | Gipc3 | Rapgef3 | Mgll | Coro2b |
| Flt1 | Kank3 | Tspan7 | Plekhg1 | Gnmt | Slc43a3 | Bace2 | Gm30938 | C1qtnf1 | Tgfbr3 |
| Cd59a | Slc9a3r2 | Armcx1 | Tmem47 | Adarb1 | Timd4 | Hspa12a | Dchs1 | Adgra2 | Cdh13 |
| Galnt18 | Adgrg3 | Gm46565 | Bcl6b | Il1a | Tcim | Tns2 | Rapgef5 | St6galnac3 | Tnfsf10 |
| Enpp6 | Gpr155 | Hmcn1 | Myzap | Gpr4 | Fbxl7 | Clec1a | Dysf | Cmtm8 | Scarf1 |
| Sox17 | Unc5a | Ptprb | Fam171a2 | Npl | Plscr4 | Sncaip | Rasgrp3 | Ephb4 | Kif26a |
| Tie1 | Serpina3g | Ltbp4 | Fam174b | Klhl4 | Dlc1 | Plxna2 | Prickle2 | Csgalnact1 | Cdh5 |
| Snai1 | Rasip1 | Dock6 | Vstm4 | Car8 | Vamp5 | Cd209b | Bmp6 | Eng | Cd55 |
| Ighg3 | Mest | Fmo1 | Arhgap29 | Chst15 | Heph | Nos3 | Hyal2 | Itga8 | Pgm5 |
| Nova2 | Bhmt | Filip1 | Stard8 | Lrrc32 | Srgap1 | Arhgap23 | Msrb3 | Caskin2 | Fscn1 |
| Slc12a5 | Pck1 | 4833403J16Rik | Adgrl2 | Apold1 | Il13ra2 | Ston1 | Gm30648 | Adcy4 | Tinagl1 |
| Tmem88 | Timp3 | Sema6a | Fat4 | Gmpr | Gm17334 | Otc | Ighv2-9-1 | Cdr2l | Amigo2 |
| Rerg | Septin4 | F2r | Lbp | Akap12 | Mcam | Pxdn | Tspan15 | Tcf7l1 | Stox2 |
| Prex2 | Twist1 | Dll1 | Ccdc80 | Rcn1 | Pcdh17 | Lama4 | Adamts7 | 1700047M11Rik | Spats2l |
| Fabp4 | Galnt15 | Dpp4 | Slco2a1 | F830016B08Rik | Epor | Mtss2 | Akr1b8 | 2810025M15Rik | Ica1 |
| Zfp503 | Slc39a8 | Jcad | Pcdhgb2 | Pecam1 | Ly6a | Loxl2 | Myo10 | Igfbp7 | Hpd |
| Aass | Efnb1 | Nr2f2 | Nid1 | Dapk2 | Fzd4 | Fcgrt | Icam2 | B3gnt3 | Gata2 |
| Plvap | 1700084E18Rik | Aqp1 | Ppic | Asah2 | Nrp2 | Tcaf2 | Ecscr | Itga1 | Sorbs3 |
| Npr2 | Lepr | Igfbp4 | Zfp521 | Ccdc9b | Zfp366 | Dpysl3 | Gm16268 | Fgf1 | Notch4 |
| Tshz3 | 2700081O15Rik | Endou | Kctd12b | Serpina3b | Bmper | Rab13 | Trpc3 | Plxnc1 | Serpina3h |
| Abat | S100a16 | Pdk4 | Snrk | Ccdc8 | Ripor3 | 2810001G20Rik | Gm10687 | Calcrl | Gm41381 |
| Fam219a | Shroom2 | Mrc1 | Chst2 | Eda2r | Amotl1 | Serpina3i | Zfp69 | Fermt2 | Rab3b |
| Hspa12b | Tal1 | D430040D24Rik | Slc24a5 | Cracr2b | Tfpi2 | Igkv2-109 | Syde1 | Dennd2a | B3galt1 |
| Shroom4 | Podxl | Gimap4 | Emilin1 | Ackr1 | Kazn | Zdhhc1 | Ptprm | Cnrip1 | Kcns3 |
| Pcdhgc3 | Cemip2 | Hoxa1 | Epn2 | Spats2 | Unc45b | Chp2 | Pard3b | Tlcd3a | Slc29a1 |
| Jam2 | Nynrin | Rilpl1 | Hspg2 | Eva1c | Pear1 | Nexn | Gm5096 | Robo1 | Mxra7 |
| Zcchc24 | Ppp1r14a | Arhgap31 | Dcbld1 | Kit | Colec12 | Rgs7bp | Hoxb5 | Stxbp1 | Rgs3 |
| Bok | Aadat | Cdc42ep5 | Gimap6 | Cadm3 | Hpgd | Mrgpre | Fabp1 | Tmtc2 | Gchfr |
| Epas1 | Prrg3 | Gm42929 | Arap3 | Klhl3 | Fcgr2b | Col4a2 | Sparc | Tspan9 | Heg1 |
| Hs3st3b1 | Igkv4-50 | Pik3c2b | Dab2ip | Trp53i11 | Tanc1 | Unc45bos | Sema3f | Upp1 | Tlr3 |
| Zeb1 | Osbpl10 | Gm11707 | Sema6b | Zbtb46 | Shisa9 | Arhgef25 | Igkv12-89 | Ighv2-6 | Dnah2os |
| Stx2 | Fcna | Ttc28 | Ccn4 | Col4a1 | Plcb4 | Pros1 | Il6st | Tat | Uaca |
| Reep1 | Slc10a6 | Nostrin | Gm33228 | Gimap8 | Inhbb | Sntb1 | Wnt9b | Ttll7 | Uroc1 |
| Prrg1 | Lbx2 | Rtl8c | Nrp1 | Fut10 | Sema4c | Ighv8-12 | Plxnd1 | Gm15675 | Prkd2 |
| Hdac7 | Zfp532 | Dmd | Gatm | Sbf2 | Mmp12 | Hlx | Ces2e | Fgd6 | Tnfaip1 |
| Vash1 | Cd163 | Fbp1 | Igkv12-41 | Calml4 | Lifr | Lpin3 | Grap |  |  |

**Table S3**. Twelve major clusters and the corresponding gene markers for main type.

| **Cell Types** | **Marker genes** | **Cluster number** | **Representative markers (Yes)** |
| --- | --- | --- | --- |
| LSECs | Cdh5 | 0,9,18,21,24,28,29 | Yes |
| LSECs | Kdr | 0,9,18,21,24,28,29 |  |
| LSECs | Bmp2 | 0,9,18,21,24,28,29 |  |
| LSECs | Flt1 | 0,9,18,21,24,28,29 |  |
| Fibroblasts/HSCs | Wt1 | 17 |  |
| Fibroblasts/HSCs | Pdgfra | 17 |  |
| Fibroblasts/HSCs | Dcn | 17 | Yes |
| Fibroblasts/HSCs | Col1a1 | 17 |  |
| Cholangiocytes | Epcam | 23 |  |
| Cholangiocytes | Sox9 | 23 |  |
| Cholangiocytes | Sorbs2 | 23 | Yes |
| Cholangiocytes | Spp1 | 23 |  |
| Hepatocytes | Mup20 | 14 | Yes |
| Hepatocytes | Alb | 14 |  |
| Hepatocytes | Ttr | 14 |  |
| Hepatocytes | Apoc1 | 14 |  |
| Plasma cells | Jchain | 26 | Yes |
| B cells | Cd79a | 4,27,31 | Yes |
| B cells | Cd79b | 4,27,31 |  |
| DCs | Runx2 | 13 |  |
| DCs | Ccr9 | 13 |  |
| DCs | Siglech | 13 | Yes |
| Kupffer cells | Csf1r | 1,5,7,8,16,19,20,25 |  |
| Kupffer cells | Adgre1 | 1,5,7,8,16,19,20,25 | Yes |
| Kupffer cells | Lyz2 | 1,5,7,8,16,19,20,25 |  |
| Kupffer cells | C1qb | 1,5,7,8,16,19,20,25 |  |
| T cells | Cd3d | 3,11,22 | Yes |
| T cells | Cd3e | 3,11,22 |  |
| T cells | Cd3g | 3,11,22 |  |
| NKs | Klrb1c | 6,10,12 |  |
| NKs | Il2rb | 6,10,12 |  |
| NKs | Nkg7 | 6,10,12 | Yes |
| Neutrophils | S100a8 | 2,15 | Yes |
| Neutrophils | S100a9 | 2,15 |  |
| Mast cells | Cpa3 | 30 | Yes |
| Mast cells | Fcer1a | 30 |  |
| Mast cells | Ms4a2 | 30 |  |

**Table S4**. Nine cell populations in fibroblasts/HSCs clustered by gene markers.

| **Cell types** | **Cluster number** | **Genes** |
| --- | --- | --- |
| aHSCs | 0, 7 | Pdgfrb |
| aHSCs | 0, 7 | Des |
| aHSCs | 0, 7 | Lum |
| iHSCs | 4, 5 | Tcf21 |
| iHSCs | 4, 5 | Smoc2 |
| iHSCs | 4, 5 | Fbln7 |
| qHSCs | None | Lrat |
| qHSCs | None | Reln |
| qHSCs | None | Gfap |
| PFs | 1, 6, 8 | Msln |
| PFs | 1, 6, 8 | Muc16 |
| PFs | 1, 6, 8 | Igfbp5 |
| Neutrophil-like fibroblasts | 2 | S100a8 |
| Neutrophil-like fibroblasts | 2 | S100a9 |
| Neutrophil-like fibroblasts | 2 | Fcgr3 |
| LSEC-like fibroblasts | 3 | Cdh5 |
| LSEC-like fibroblasts | 3 | Kdr |
| LSEC-like fibroblasts | 3 | Bmp2 |

**Table S5**. ICAM, JAM, CCL, CHEMERIN, and GAS pathways with contributing ligands in aHSCs and receptors in neutrophils, Kupffer cells, and B cells.

| **Pathways** | **Ligands** | **Receptors** | | |
| --- | --- | --- | --- | --- |
|  | **aHSCs** | **Neutrophils** | **Kupffer cells** | **B cells** |
| **CCL** | Ccl2 | Ccr1 | Ccr1 | Ccr1 |
|  | Ccl3 | Ccr2 | Ccr2 | Ccr2 |
|  | Ccl4 | Ccr5 | Ccr5 | Ccr5 |
|  | Ccl5 | Ccr7 | Ccr7 | Ccr7 |
|  | Ccl6 | Ackr1 | Ackr1 | Ackr1 |
|  | Ccl7 |  |  |  |
|  | Ccl9 |  |  |  |
|  | Ccl19 |  |  |  |
|  | Ccl27a |  |  |  |
| **ICAM** | Icam1 | **Itgb2** | **Itgb2** | **Itgb2** |
|  | Icam2 | **Itgam** | **Itgam** | **Itgam** |
|  |  | **Itgal** | **Itgal** | **Itgal** |
|  |  | Itgax | Itgax | Itgax |
|  |  | Spn | Spn | Spn |
|  | Icam1 | **Itgb2** | **Itgb2** | **Itgb2** |
|  | Icam2 | **Itgam** | **Itgam** | **Itgam** |
|  |  | **Itgal** | **Itgal** | **Itgal** |
| **JAM** | Jam2 | Jam2 | Jam2 | Jam2 |
|  | Jam3 | Jam3 | Jam3 | Jam3 |
|  | F11r/Jam1 | F11r | F11r | F11r |
|  |  | **Itgam** | **Itgam** | **Itgam** |
|  |  | **Itgal** | **Itgal** | **Itgal** |
|  |  | Itgb1 | Itgb1 | Itgb1 |
|  |  | **Itgb2** | **Itgb2** | **Itgb2** |
|  |  | Itgav | Itgav | Itgav |
| **CHEMERIN** | Rarres2 | Cmklr1 | Cmklr1 | Cmklr1 |
| **GAS** | Gas6 | Axl | Axl | Axl |
|  |  | Mertk | Mertk | Mertk |
|  | | | | |
|  | **Gene list for aHSCs** | **Gene list for Neutrophils** | **Gene list for Kupffer cells** | **Gene list for B cells** |
|  | Ccl2 | Ccr1 | Ccr1 | Ccr1 |
|  | Ccl3 | Ccr2 | Ccr2 | Ccr2 |
|  | Ccl4 | Ccr5 | Ccr5 | Ccr5 |
|  | Ccl5 | Ccr7 | Ccr7 | Ccr7 |
|  | Ccl6 | Ackr1 | Ackr1 | Ackr1 |
|  | Ccl7 | Itgax | Itgax | Itgax |
|  | Ccl9 | Spn | Spn | Spn |
|  | Ccl19 | Itgb2 | Itgb2 | Itgb2 |
|  | Ccl27a | Itgam | Itgam | Itgam |
|  | Icam1 | Itgal | Itgal | Itgal |
|  | Icam2 | Jam2 | Jam2 | Jam2 |
|  | Jam2 | Jam3 | Jam3 | Jam3 |
|  | Jam3 | F11r | F11r | F11r |
|  | F11r/Jam1 | Itgb1 | Itgb1 | Itgb1 |
|  | Rarres2 | Itgav | Itgav | Itgav |
|  | Gas6 | Cmklr1 | Cmklr1 | Cmklr1 |
|  |  | Axl | Axl | Axl |
|  |  | Mertk | Mertk | Mertk |

**Table S6**. Antibodies used in the study.

| **Antibody name** | **Supplier** | **Catalog No.** | **Usage** | **Dilution/dosage** |
| --- | --- | --- | --- | --- |
| TGF-β1 | Abcam | ab215715 | IHC | 1:200 |
| α-SMA | PL Laboratories | PL0300189 | IHC | 1:500 |
| Col-I | PL Laboratories | PL0306785 | IHC | 1:500 |
| Des | Proteintech | 16520-1-AP | IF | 1:1000 |
| Icam1 | Proteintech | 10020-1-AP | IF | 1:2000 |
| S100a8+S100a9 | Abcam | ab288715 | IF | 1:4000 |
| Itgal/CD11a | Affinity | DF6525 | IF | 1:200 |
| Itgam/CD11b | Abcam | ab133357 | IF | 1:4000 |
| Itgb2/CD18 | Affinity | DF6896 | IF | 1:100 |
| PE mouse anti-human CD29 | BD Biosciences | 555443 | FACS | 10 μL |
| PE mouse anti-human CD34 | BD Biosciences | 555822 | FACS | 10 μL |
| PE mouse anti-human CD45 | BD Biosciences | 560975 | FACS | 2.5 μL |
| PE mouse anti-human CD73 | BD Biosciences | 550257 | FACS | 10 μL |
| PE mouse anti-human CD90 | BD Biosciences | 555596 | FACS | 2.5 μL |
| PE mouse anti-human CD105 | BD Biosciences | 560839 | FACS | 2.5 μL |
| PE mouse anti-human CD117 | BD Biosciences | 555714 | FACS | 2.5 μL |
| PE mouse anti-human HLA-DR | BD Biosciences | 555812 | FACS | 10 μL |
| PE mouse IgG1 | BD Biosciences | 555749 | FACS | 10 μL |
| PE mouse IgG2a | BD Biosciences | 555574 | FACS | 10 μL |

**Note:** IHC, immunohistochemistry; IF, immunofluorescence; FACS, fluorescence-activated cell sorting.

**Table S7.** Primers used for PCR analysis.

| **Gene name** | **Species/host** | **Sequence (5’→3’)** | **Usage** |
| --- | --- | --- | --- |
| oIMR7338-F | Mouse | CTAGGCCACAGAATTGAAAGATCT | Internal positive control, PCR |
| oIMR7339-R | Mouse | GTAGGTGGAAATTCTAGCATCATCC |  |
| HBV1.28seq-F | Virus | CGCCTCGCAGACGCAGAT | HBV product, PCR |
| HBV1.28seq-R | Virus | CGAACCACTGAACAAATGGCACTA |  |
| Col1a1-F | Mouse | GCTGGTCCCCGAGGAAACAAT | Col-I, qRT-PCR |
| Col1a1-R | Mouse | CACGTTCACCAGGCATTCCC |  |
| X-universal-F | Virus | CCGTCTGTGCCTTCTCATCT | HBV product, qRT-PCR |
| X-universal-R | Virus | TAATCTCCTCCCCCAACTCC |  |
| mGAPDH-F | Mouse | TGCACCACCAACTGCTTAG | Internal control, qRT-PCR |
| mGAPDH-R | Mouse | GGATGCAGGGATGATGTTC |  |
| mActa2-F | Mouse | cGAcactgctgacagaggcacca | α-SMA, qRT-PCR |
| mActa2-R | Mouse | ATAGGCACGTTGTGAGTCACACCA |  |
| mTgfb1-F | Mouse | ACTGGAGTTGTACGGCAGTG | TGF-β1, qRT-PCR |
| mTgfb1-R | Mouse | GGGGCTGATCCCGTTGATTT |  |
| β-actin-F | Human | ATCAAGATCATTGCTCCTCCTG | β-actin, qRT-PCR |
| β-actin-R | Human | GTCATACTCCTGCTTGCTGAT |  |
| Icam1-F | Human | TTGGGCATAGAGACCCCGTT | Icam1, qRT-PCR |
| Icam1-R | Human | GCACATTGCTCAGTTCATACACC |  |

**Note:** F, forward; R, reverse; qRT-PCR, quantitative real-time polymerase chain reaction.


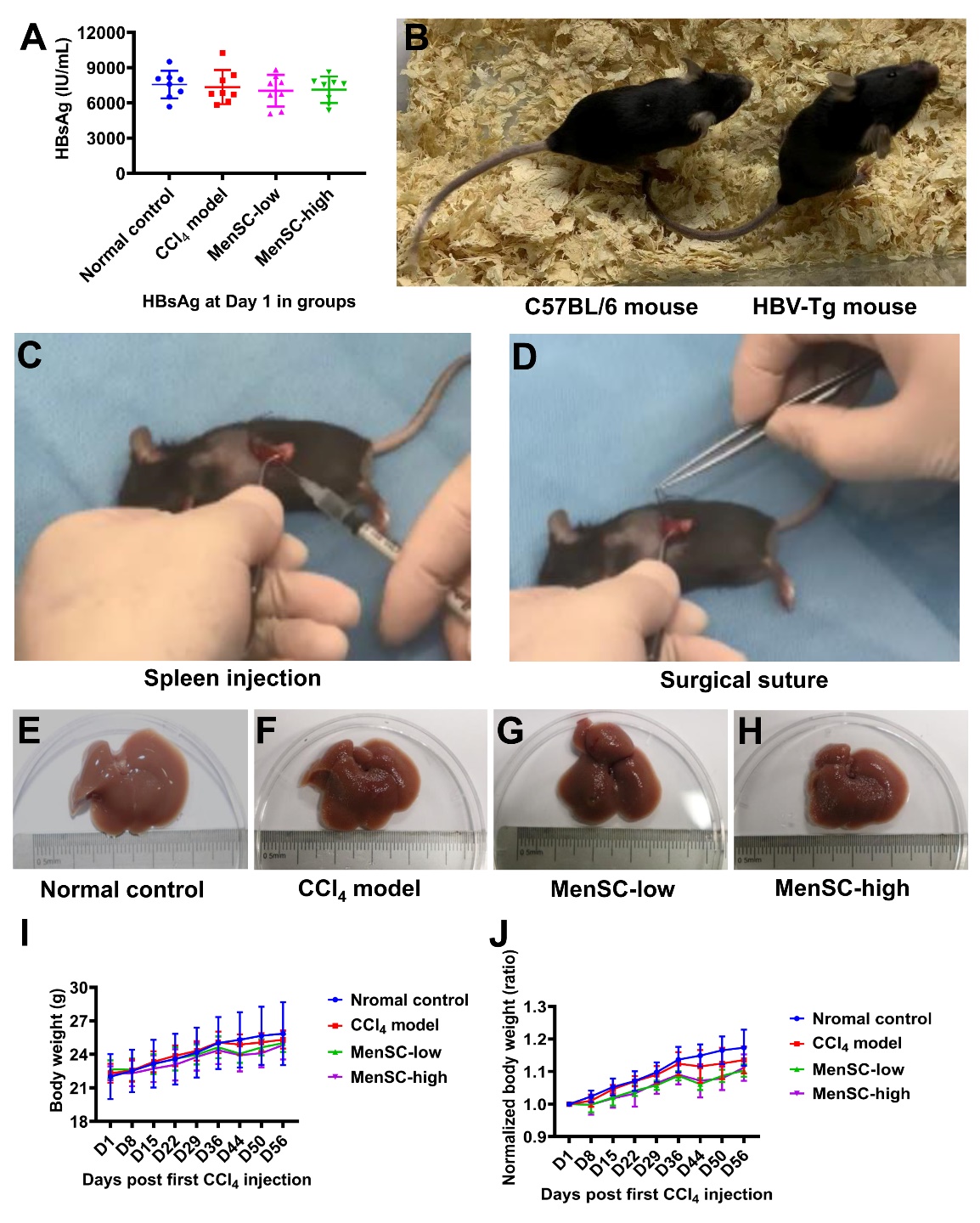


**Figure S1**. (A), The enrolled mice of hepatitis B surface antigen (HBsAg) using serum test, the average of HBsAg are not significant in normal control group (N = 8), CCl_4_ model group (N = 8), MenSC-low group (N = 8), and MenSC-high group (N = 8). (B), The phenotype of HBV-Tg and C57BL/6 mice. The respective spleen injection (C) and surgical suture (D) for MenSC transplantation. The respective HBV-Tg mouse livers in normal control group (E), CCl_4_ model group (F), MenSC-low group (G), and MenSC-high group (H). Through analysis of the body weight (I) and normalized body weight (J) at different groups, there is no significant difference in body weight between the CCl_4_ model, MenSC-low and MenSC-high groups.


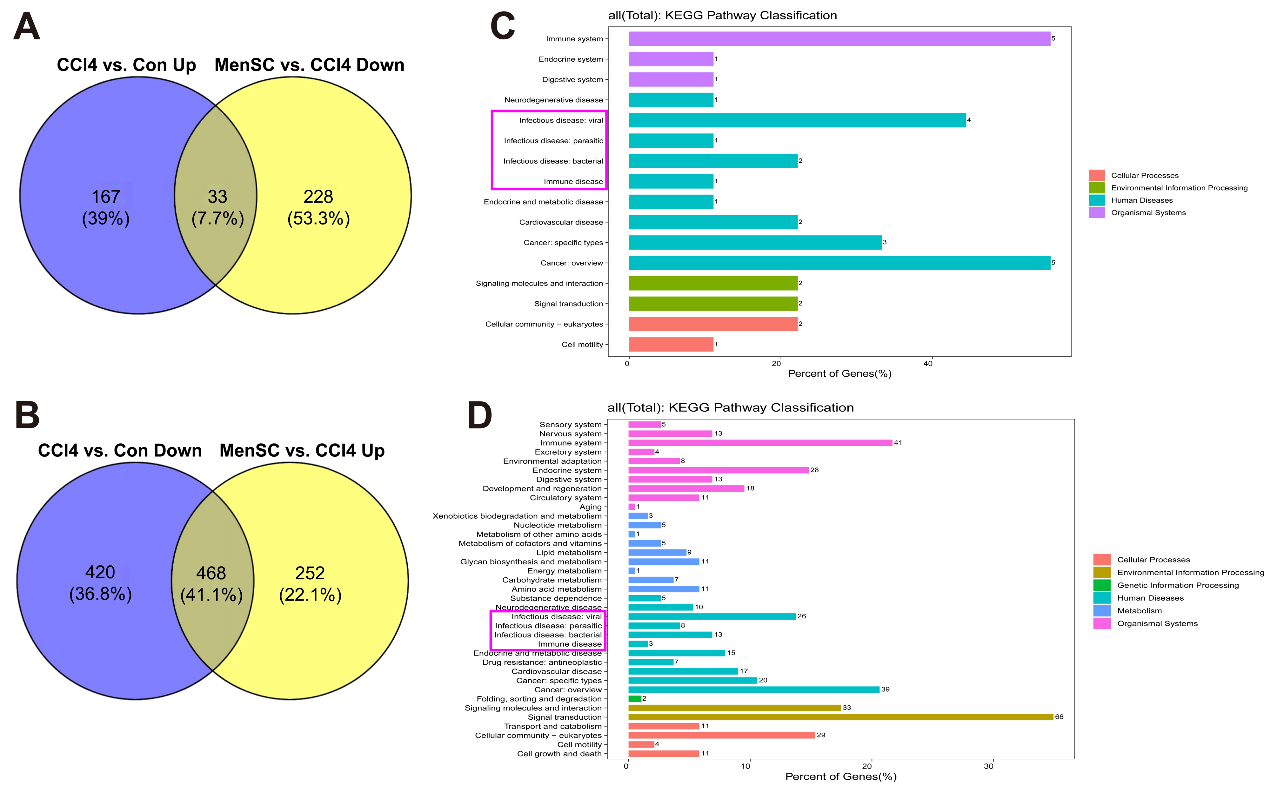


**Figure S2**. According to the RNA-seq analysis, 33 common genes (A) in “CCl_4_ vs. Con up” and “MenSC vs. CCl_4_ down” and 468 common genes (B) in “CCl_4_ vs. Con down” and “MenSC vs. CCl_4_ up”. Infectious diseases and immune-related diseases were in total KEGG pathway based on the 33 common genes (C) and 468 common genes (D).


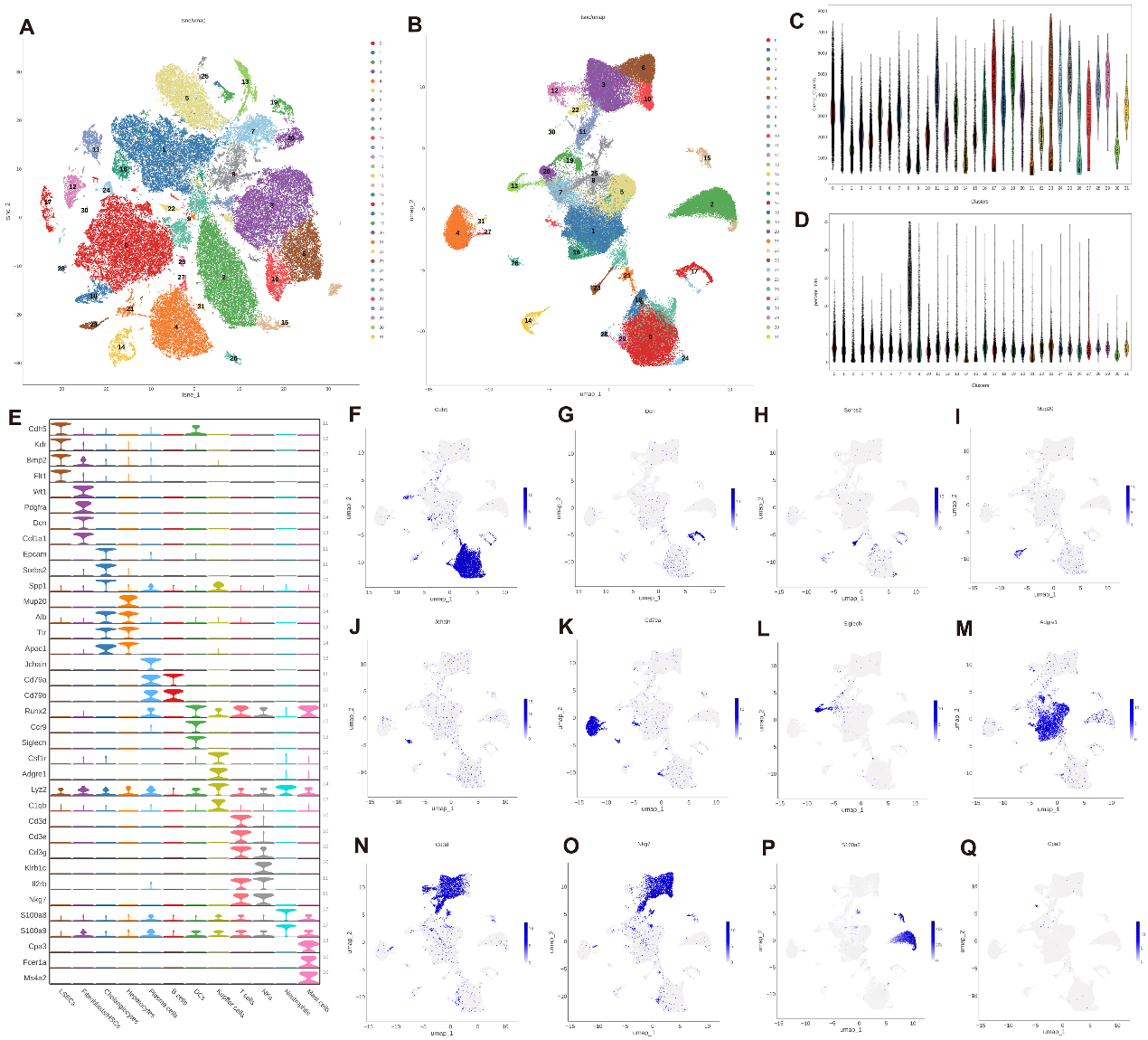


**Figure S3**. The initially visual diagrams of t-SNE (A) and UMAP (B) were presented in the 32 clusters. (C), the Gene_Counts_RNA profile for 32 cell clusters. (D), the percentage of mitochondrial genes in the dataset. (E), 12 major cell types in violin plot along with the corresponding gene markers: LSECs (*Cdh5, Kdr, Bmp2, Flt1*), fibroblasts/HSCs (*Wt1, Pdgfra, Dcn, Col1a1*), cholangiocytes (*Epcam, Sox9, Sorbs2, Spp1*), hepatocytes (*Alb, Ttr, Mup20, Apoc1*), plasma cells (*Jchain*), B cells (*Cd79a, Cd79b*), DCs (*Runx2, Ccr9, Siglech*), Kupffer cells (*Csf1r, Adgre1, Lyz2, C1qb*), T cells (*Cd3d, Cd3e, Cd3g*), NKs (*Il2rb, Nkg7, Klrb1c*), neutrophils (*S100a8, S100a9*), and mast cells (*Cpa3, Fcer1a, Ms4a2*). Representative markers for each cluster with Feature Plot were *Cdh5* (F), *Dcn* (G), *Sorbs2* (H), *Mup20* (I), *Jchain* (J), *Cd79a* (K), *Siglech* (L), *Adgre1* (M), *Cd3d* (N), *Nkg7* (O), *S100a8* (P), and *Cpa3* (Q).


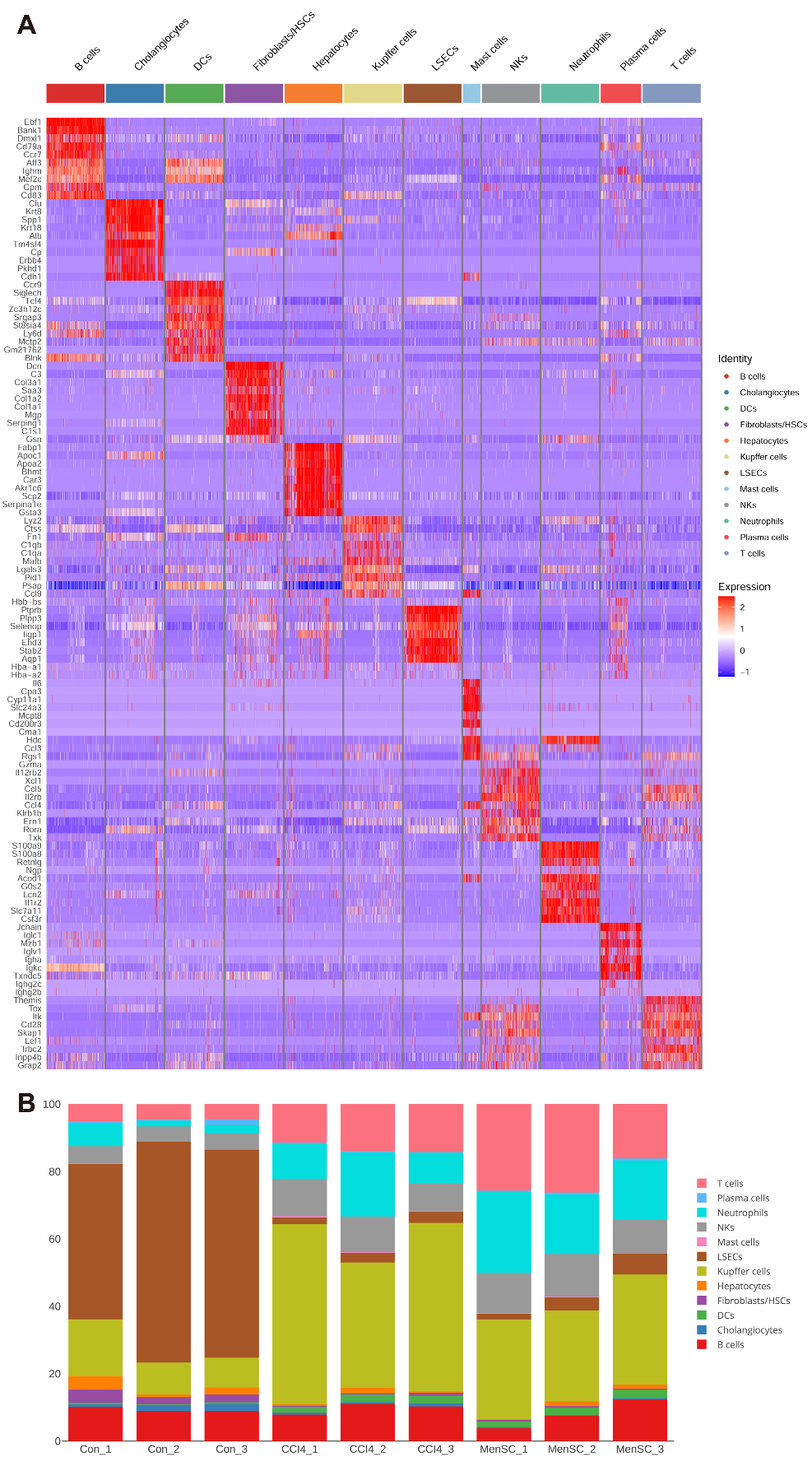


**Figure S4**. (A), The heatmap of top 10 markers of differentially expressed genes (DEGs) in 12 main types. (B), The cell proportion of each sample in the Con group, CCl_4_ group, and MenSC group.


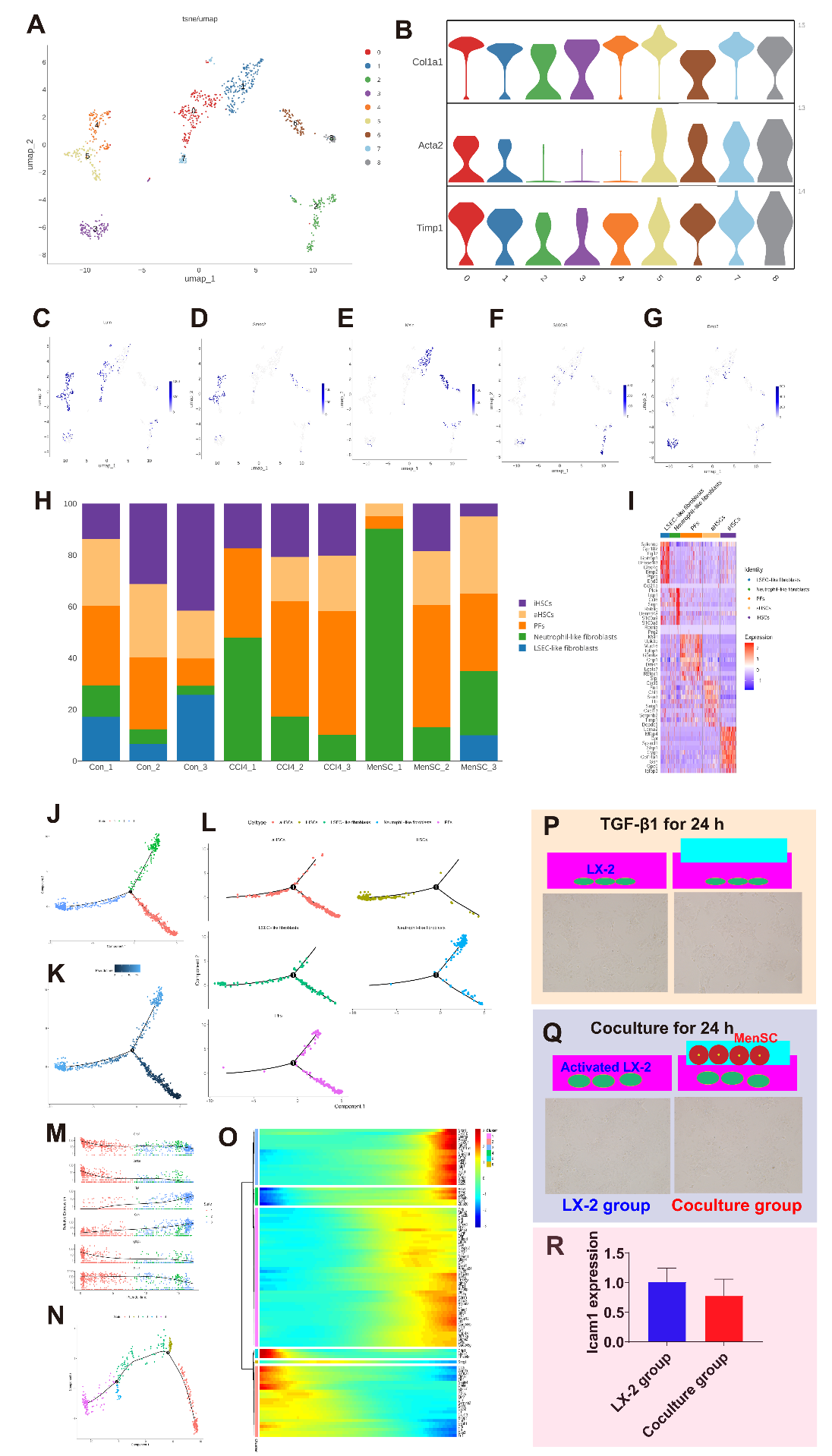


**Figure S5**. (A), unsupervised clustering analysis of UMAP in identified nine cell populations. (B), classic markers including *Col1a1*, *Timp1*, and *Acta2* in nine major clusters of the fibroblasts/HSCs proportions in violin plot. The representative markers of *Lum* (C), *Smoc2* (D), *Msln* (E), *S100a8* (F), and *Bmp2* (G) for each cluster with Feature Plot. (H), the cell proportion of aHSCs, iHSCs, PFs, neutrophil-like fibroblasts, and LSEC-like fibroblasts with each sample in the Con group, CCl_4_ group, and MenSC group. (I), the heatmap for top 10 markers of differentially expressed genes (DEGs) in aHSCs, iHSCs, PFs, neutrophil-like fibroblasts, and LSEC-like fibroblasts. (J), different stages in the Monocle2. The aHSCs and PFs are major in stage 1, Neutrophil-like fibroblasts are major in stage 2, iHSCs and PFs are major in stage 3, LSEC-like fibroblasts are exist at all stages. (K), Pseudotime analysis in subpopulations of fibroblasts/HSCs. (L), Pseudotime analysis in individual subpopulations of fibroblasts/HSCs. (M), with the time course, the most upping genes for the fibroblasts/HSCs proportions were *Dpt* and *Gsn*, and the most downing genes *Crip*, *Dmkn*, *Igfbp5*, and *Saa3*. (N), aHSCs and iHSCs can be divided into 5 stages in the Monocle2. (O), six sub-subpopulations and their relative gene expressions with the time process. (P), Recombinant TGF-β1 stimulated LX-2 cells for 24 h to activated HSCs. (Q), aHSC cocultured with MenSC for 24 h *in vitro*, and individual LX-2 cells named as LX-2 group, MenSC and LX-2 in the transwell named as coculture group. (R), The *Icam1* expression of LX-2 cells in LX-2 group and coculture group was assessed by qRT-PCR.


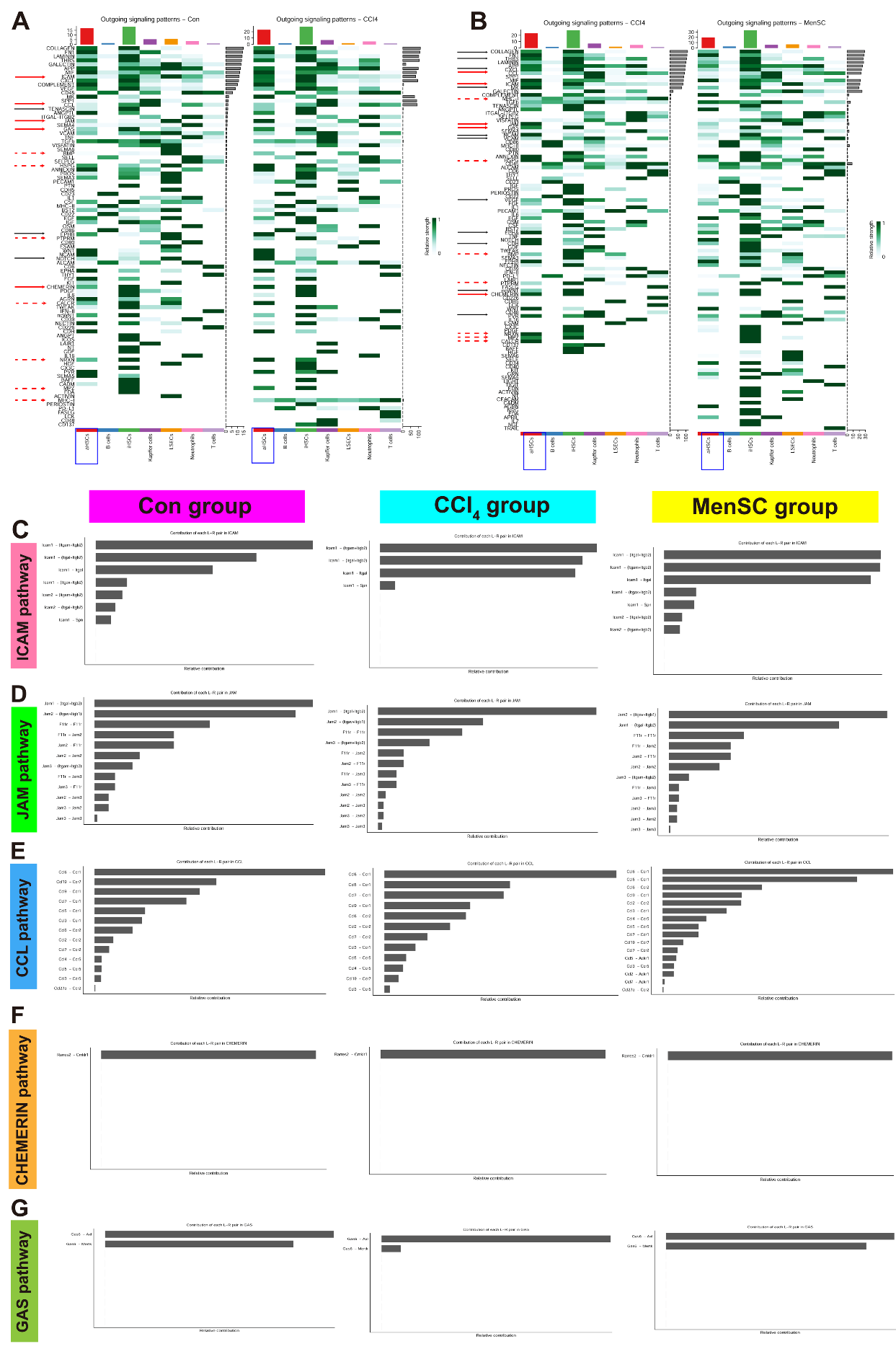


**Figure S6**. Outgoing signaling pathways of aHSCs, iHSCs, T cells, B cells, Kupffer cells, neutrophils, and LSECs in CCl_4_ group vs. Con group (A) and MenSC group vs. CCl_4_ group (B). The output signal of aHSC is higher in the CCl_4_ groups than the Con group and the MenSC group. Among these, 15 signal pathways were stronger than Con group in CCl_4_ group (A), 23 signals were weaker than CCl_4_ group in MenSC group (B). Some common pathways (marked with red dashed line) were found to be consistent, but there are neither no expression or no signals targeting neutrophils/Kupffer cells/B cells. Five signal channels were selected including ICAM, JAM, CCL, CHEMERIN, and GAS (marked with red font). The relative contribution of ligands in aHSCs and receptors in neutrophils/Kupffer cells/B cells for ICAM pathway (C), JAM pathway (D), CCL pathway (E), CHEMERIN pathway (F), and GAS pathway (G) in the Con group, CCl_4_ group, and MenSC group.


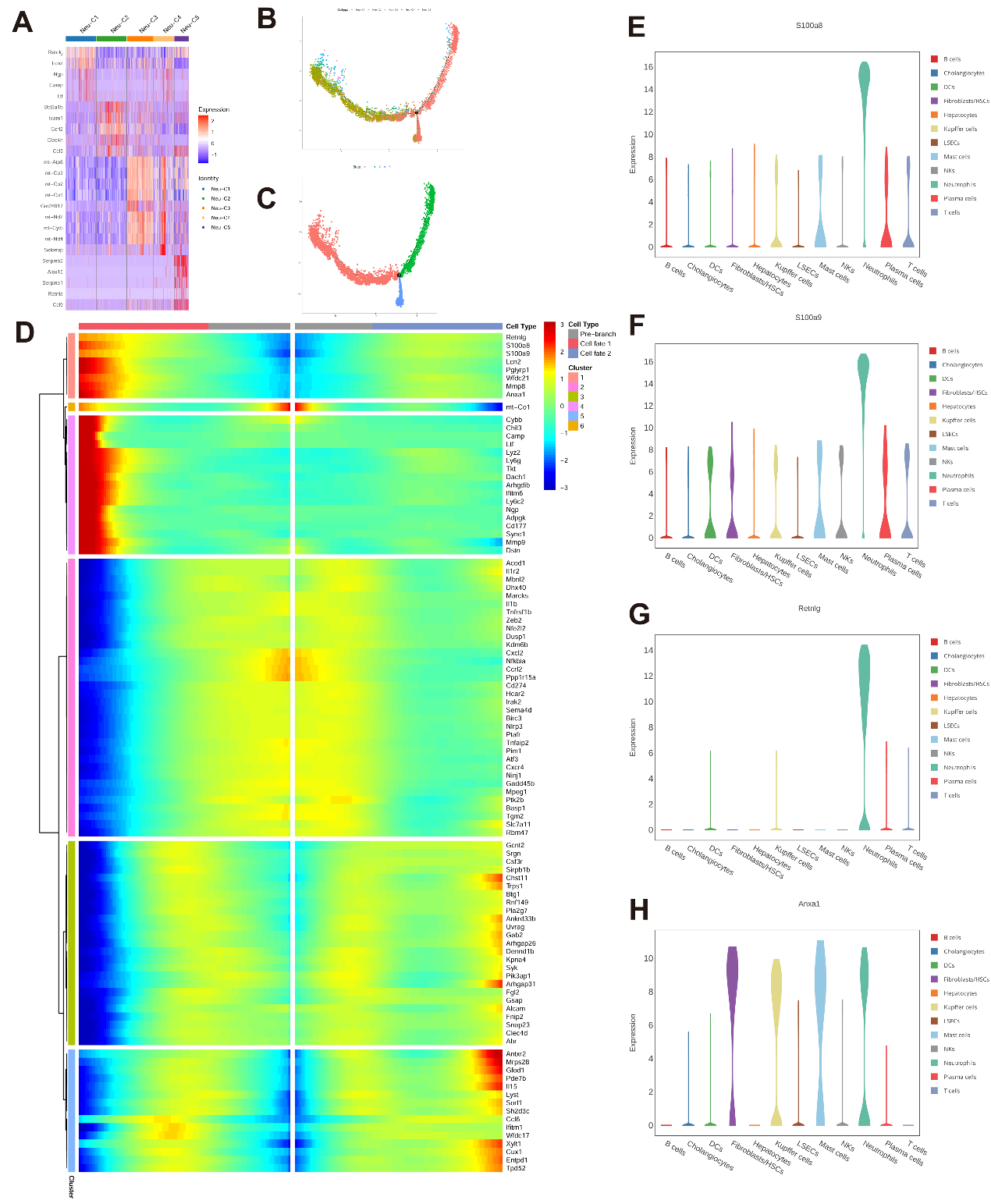


**Figure S7**. (A), The heatmap of top 5 markers of DEGs in subgroup of neutrophils, including Neu-C1, Neu-C2, Neu-C3, Neu-C4, and Neu-C5. (B), Pseudotime analysis found Nue-C1 and Neu-C2 clusters formed a relatively complete timeline. (C), Neu-C2 was mainly distributed in stage 1, and the Neu-C1 was mainly distributed in stage 2 and stage 3. (D), eight of top 10 genes were able to cluster together from pre-branch to cell-fate 1/2, including *Anxa1*, *Retnlg*, *Wfdc21*, *Mmp8*, *Pglyrp1*, *S100a8*, *S100a9*, and *Lcn2*. Relative expression profiles of *S100a8* (E), *S100a9* (F), and *Retnlg* (G) in the main types were verified. (H), The relative expression profile of *Anxa1*.
